# Src regulates amino acid-mediated mTORC1 activation by disrupting GATOR1-Rag GTPase interaction

**DOI:** 10.1038/s41467-018-06844-4

**Published:** 2018-10-19

**Authors:** Rituraj Pal, Michela Palmieri, Arindam Chaudhury, Tiemo Jürgen Klisch, Alberto di Ronza, Joel R. Neilson, George G. Rodney, Marco Sardiello

**Affiliations:** 1 Department of Molecular and Human Genetics, Baylor College of Medicine, Jan and Dan Duncan Neurological Research Institute, Texas Children’s Hospital, Houston, TX 77030 USA; 2 Department of Molecular Physiology and Biophysics, Baylor College of Medicine, One Baylor Plaza, Houston, TX 77030 USA

## Abstract

The mechanistic target of rapamycin complex 1 (mTORC1) regulates cell survival and autophagy, and its activity is regulated by amino acid availability. Rag GTPase-GATOR1 interactions inhibit mTORC1 in the absence of amino acids, and GATOR1 release and activation of RagA/B promotes mTORC1 activity in the presence of amino acids. However, the factors that play a role in Rag-GATOR1 interaction are still poorly characterized. Here, we show that the tyrosine kinase Src is crucial for amino acid-mediated activation of mTORC1. Src acts upstream of the Rag GTPases by promoting dissociation of GATOR1 from the Rags, thereby determining mTORC1 recruitment and activation at the lysosomal surface. Accordingly, amino acid-mediated regulation of Src/mTORC1 modulates autophagy and cell size expansion. Finally, Src hyperactivation overrides amino acid signaling in the activation of mTORC1. These results shed light on the mechanisms underlying pathway dysregulation in many cancer types.

## Abstract (web summary)

The growth-promoting activity of mTORC1 is regulated by amino acid availability via the Rag GTPases. Here, the authors demonstrate Src-dependent control of cell size and autophagy through disruption of the Rag GTPase–GATOR1 complex and mTORC1 activation at the lysosomal surface.

## Introduction

The mechanistic target of rapamycin (mTOR) protein kinase is a large catalytic subunit that exists in at least two distinct complexes, mTORC1 and mTORC2, which regulate cell growth and proliferation and are often dysregulated in disease^1–3^. mTORC1 integrates diverse inputs, including signals associated with growth factor activity, cellular energy levels, and amino acid availability to coordinate cell metabolism^1,2^. Activation of mTORC1 promotes cell growth by stimulating anabolic processes such as transcription, ribosomal biogenesis and translation^4–6^, whereas mTORC1 inactivation promotes catabolic processes such as autophagy to meet energy demands in conditions of nutrient paucity^6–8^. Many pathways that signal to mTORC1 converge on TSC1/2, a heterodimeric tumor suppressor that negatively regulates Rheb guanosine triphosphatase (Rheb GTPase), which is an essential activator of mTORC1^2^. Unlike many other inputs, amino acids regulate mTORC1 activity independently of TSC2 via the Rag GTPases, which form heterodimeric complexes comprised of RagA or RagB bound to RagC or RagD^9^.

Amino acid signaling to mTORC1 requires a lysosomal membrane-associated machinery that consists of the Rag GTPases, the Ragulator complex, and the vacuolar adenosine triphosphatase (V-ATPase)^10,11^. In response to amino acids, the guanine nucleotide exchange factor (GEF) activity of Ragulator is promoted toward RagA and RagB that, when guanosine triphosphate (GTP)-loaded, recruit mTORC1 to the lysosomal surface. mTORC1 may then be fully activated by its potent and essential direct activator, Rheb^12,13^. Upon removal of amino acids, Rag GTPase-activating protein (GAP) complex GATOR1 induces the Rag dimers to switch into an inactive conformation containing guanosine diphosphate (GDP)-bound RagA/B, thereby releasing mTORC1 from the lysosomal surface, which in turn results in the inactivation of mTORC1^10^. Upon addition of amino acids, GATOR2, a positive regulator of the Rags, initiates GTP loading of RagA/B via inhibition of GATOR1^10,14,15^. Amino acid stimulation promotes dissociation of GATOR1 from the Rags^9^, thus establishing GATOR1 repression of the Rags as a major regulatory axis of mTORC1 activation in response to amino acids.

Owing to the central role of mTORC1 in the control of cell growth and metabolism, numerous studies have investigated regulation of mTORC1 by oncogenes and tumor suppressors that are implicated in familial or sporadic forms of cancer^16,17^. Among these, the non-receptor tyrosine kinase, Src, is an oncogene of paramount importance. Several studies have identified Src as a critical component of the signal transduction pathways that control cancer cell growth^18,19^.

Here, we show that Src is a critical regulator of amino acid-mediated activation of mTORC1. We demonstrate that Src acts upstream of the Rag GTPases by promoting dissociation of GATOR1 from the Rags, thereby modulating mTORC1 recruitment and activation at the lysosomal surface. Accordingly, we show that amino acid-mediated regulation of Src/mTORC1 modulates autophagy and cell size expansion. In addition, we show that Src hyperactivation overrides amino acid signaling in the activation of mTORC1, a finding that could shed light on the mechanisms underlying pathway dysregulation in many cancer types.

## Results

### Src regulates amino acid-mediated mTORC1 activation

**Fig. 1:**
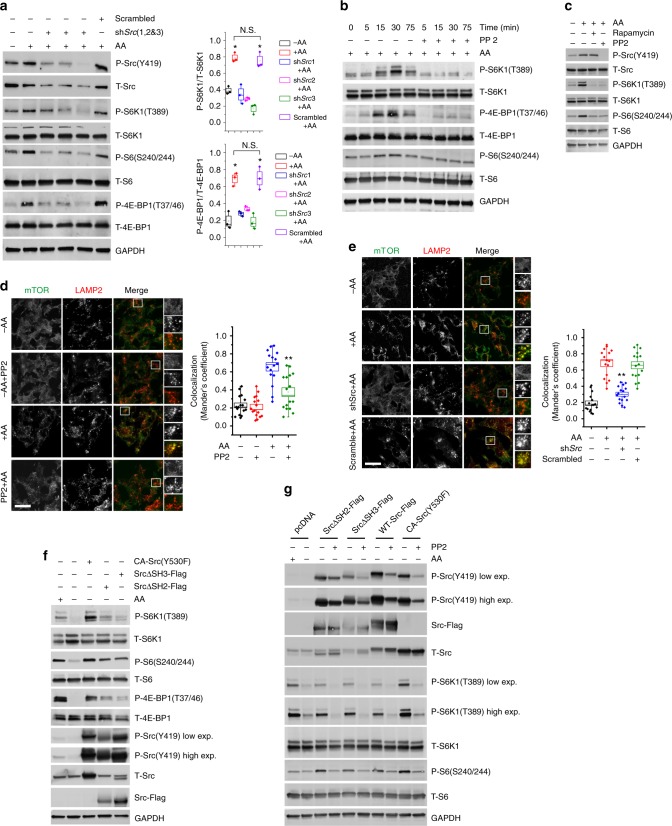
Src is necessary and sufficient for amino acid-mediated activation of mTORC1. **a** SH-SY5Y cells were treated with *SRC* shRNAs, starved, and stimulated with amino acids for 30 min. Lysates were probed with antibodies as indicated. Box plots represent SE of *n* = 3 independent experiments. *p < 0.05. N.S. represents “not significant”. **b** SH-SY5Y cells were starved and treated with vehicle (DMSO) or PP2 (10 μM) for the last 2 h of starvation prior to stimulation with amino acids at several time points. Lysates were probed with antibodies as indicated. **c** SH-SY5Y cells were starved and treated with vehicle (DMSO) or PP2 (10 μM), or rapamycin (600 nM) for the last 2 h of starvation and then stimulated with amino acids (30 min). Lysates were probed with antibodies as indicated. **d** SH-SY5Y cells were treated as in **b** but with a single 30 min amino acid stimulation prior to immunofluorescence labeling of endogenous LAMP2 (red) and mTOR (green). **e** SH-SY5Y cells were treated as in **a** and labeled and analyzed as in **d**. Bar indicates 60 μm. Representative cells are shown where yellow or orange pixels indicate co-localization in the merged images. In all images in **d** and **e** insets show selected fields that were magnified by a factor of 4. Scale bar indicates 60 μm. Quantified data represent mean co-localization (Mander’s coefficient) of mTOR and LAMP2 in at least 15 cells for each condition. Box plots represent SE of *n* = 3 independent experiments. ** indicates significant differences (**p < 0.01) between all conditions in each group. **f** SH-SY5Y cells, transiently transfected with Y530F-Src, SrcΔSH3, or SrcΔSH2, were starved with amino acids (kept in dialyzed serum for 4 h). Cells were then lysed and immunoblot analyses were used to measure the levels of the indicated proteins and phosphorylation states. **g** SH-SY5Y cells were transiently transfected with the indicated Src constructs and starved as in **f**. Cells were treated with PP2 (10 μM), where indicated, for the last 2 h of starvation prior to lysis and immunoblot analyses. GAPDH was used as a loading control in all immunoblot assays. Statistical differences between groups in **a**, **d**, and **e** were determined using ANOVA with Tukey’s post hoc test

We hypothesized that Src is involved in amino acid-mediated regulation of mTORC1, and set out to test this hypothesis by first defining the conditions in which optimal stimulation of mTORC1 by amino acids is observed in cultured SH-SY5Y cells and mouse embryonic fibroblasts (MEFs). A time-course analysis showed that consistent activation of mTORC1 occurs 30 min after amino acid replenishment in both cell lines (Supplementary Fig. 1a,b). We therefore used a 30-min period of amino acid stimulation in subsequent studies. Consistent with previous reports^12^, we found that, in SH-SY5Y cells, mTOR predominantly co-localized with LAMP2-positive lysosomal vesicles upon amino acid stimulation, whereas amino acid starvation promoted cytosolic localization of mTOR with minimal amounts on the lysosomal surface (Supplementary Fig. 1c). To investigate whether amino acid activation of mTORC1 depends on Src function, we suppressed Src activity by using either short hairpin RNAs (shRNAs) targeting *SRC*, or Src pharmacological inhibitors (dasatinib^20^ and PP2^21^). Both genetic and pharmacological inhibition of Src activity decreased amino acid-induced activation of mTORC1 with no changes in total mTOR protein levels (Fig. 1a, b and Supplementary Fig. 1d-f). While PP2 treatment suppressed amino acid-mediated activation of both Src and mTORC1, rapamycin only inhibited mTORC1 activity (Fig. 1c), indicating that Src is an upstream regulator of mTORC1. Confocal microscopy experiments showed that mTOR translocation from the cytosol to lysosomes upon amino acid stimulation was abolished by pharmacological or genetic inhibition of Src activity (Fig. 1d, e). Thus, Src acts as an essential upstream factor that controls amino acid-dependent localization and activation of mTORC1.

We next tested whether Src activity per se is sufficient for the activation of mTORC1, irrespective of amino acid availability. From the N-terminus to C-terminus, Src is comprised of a myristoyl group attached to a unique SH4 domain (U) followed by SH3 and SH2 domains, an SH2-kinase linker, a protein–tyrosine kinase domain (also known as SH1 domain), and a C-terminal regulatory segment^19,22^. Mutations at Src Y530 or deletion of Src SH2/SH3 domains result in constitutive activation of Src^23^ as evidenced by autophosphorylation at Y419^22^ (Supplementary Fig. 1g,h). Wild-type Src (WT-Src-Flag) and constitutively active Src constructs obtained by mutating Y530 to phenylalanine (Y530F-Src)^24,25^ or by deleting the SH3 or SH2 domain (SrcΔSH3-Flag and SrcΔSH2-Flag) were expressed in cultured cells. All constructs significantly increased mTORC1 activity in normal growth conditions, which was attenuated by PP2-mediated Src inhibition (Supplementary Fig. 1i, j). Interestingly, transfection of constitutively active Src resulted in insensitivity of mTORC1 to amino acid deprivation, which was reversed by pharmacological inhibition of Src (Fig. 1f, g). These results suggest that the kinase activity of Src is both necessary and sufficient to induce mTORC1 activation in the context of the amino acid-sensing machinery.

### Src regulates mTORC1 at lysosomes independently of TSC2

**Fig. 2:**
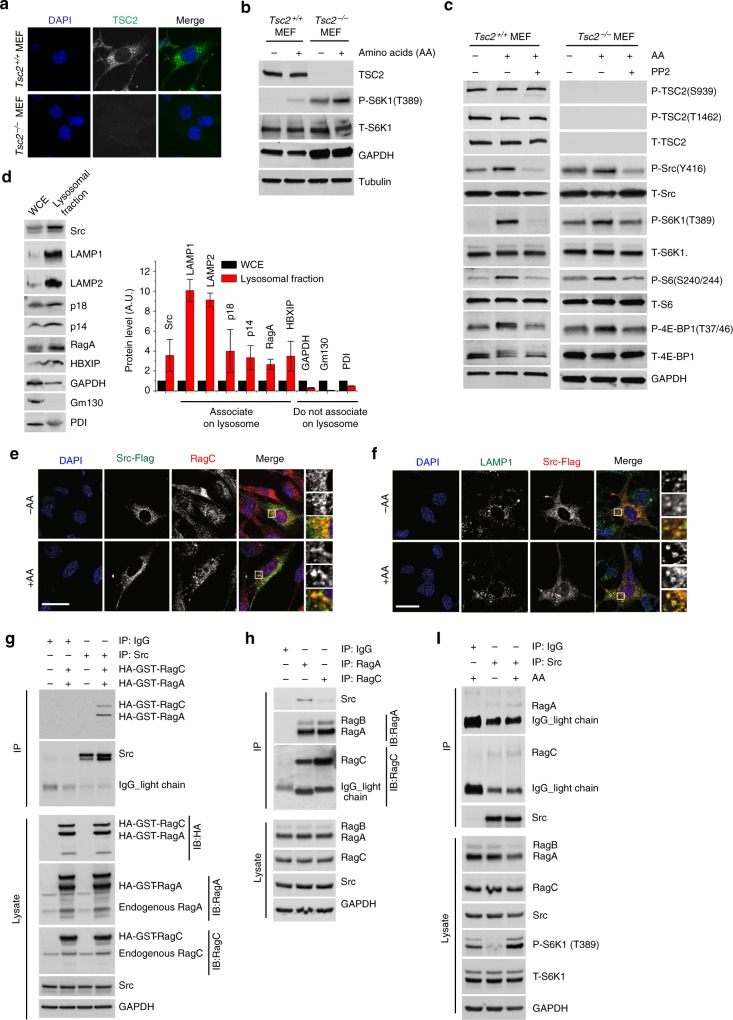
Src regulates mTORC1 activity independently of TSC2. **a** WT (*Tsc2*^*+/+*^*)* and TSC2 null *(Tsc2*^*−/−*^*)* MEF cells were coimmunostained for DAPI (blue) and TSC2 (green). Bar indicates 40 μm. **b** Amino acid-starved *Tsc2*^*−/−*^ MEFs showed significantly higher activity of mTORC1 compared to *Tsc2*^*+/+*^ MEFs. Activation was further potentiated upon stimulation with amino acids. Lysates were probed with antibodies as indicated. **c** *Tsc2*^*+/+*^ and *Tsc2*^*−/−*^ MEFs cells were starved and treated with vehicle (DMSO) or PP2 (10 μM) for the last 2 h of starvation and then stimulated with amino acids (30 min). Lysates were probed with antibodies as indicated. **d** A lysosome-enriched cell fraction was isolated from SH-SY5Y cells and used to perform immunoblot analyses to measure the levels of the indicated proteins. LAMP1, LAMP2, p18, p14, RagA, and HBXIP are known lysosome-associated protein^10^. GM130 and PDI are used as Golgi and ER markers, respectively^47,48^. WCE stands for whole-cell extract. Quantified data represent means ± SEM of *n* = 2–3 independent experiments. **e**, **f** MEF cells were transfected to express Src, starved and stimulated with amino acids (30 min) prior to immunofluorescent labeling of RagC (red) and Src (green) in **e** and LAMP1 (green) and Src (red) in **f**. Representative cells are shown where yellow or orange pixels indicate co-localization in the merged images. In all images, insets show selected fields that were magnified by a factor of 4. Bar indicates 40 μm. **g** HEK cells, transiently transfected with HA-GST-RagA and HA-GST-RagC, were lysed and co-IP analyses were performed to test interaction of Src with the Rags. Immunoblot analyses were used to measure the levels of the indicated proteins. **h** HEK cells were lysed and coIP analyses were performed to test interaction of Src with the Rags. **i** HEK cells were starved prior to amino acid stimulation (30 min). Cells were lysed and coIP analyses were performed. IgG antibody pulldown was performed as a negative control in **g** to **i**. GAPDH and tubulin were used as a loading control in immunoblot assays

To investigate whether Src-dependent amino acid regulation of mTORC1 funnels through TSC2, a known key negative regulator of mTORC1 activity^2^, we used *Tsc2*^*−/−*^ MEFs (Fig. 2a) where the activity of mTORC1 is significantly higher than in WT MEFs (*Tsc2*^*+/+*^) independent of amino acid availability. Growth factors such as insulin regulate mTORC1 activity through the Akt-TSC2 axis^2^. While in *Tsc2*^*+/+*^ MEFs insulin-mediated stimulation of mTORC1 activity was abolished upon treatment with the Akt inhibitor MK2206^26^, *Tsc2*^−^^*/−*^ MEFs were insensitive to both insulin deprivation and Akt inhibition (Supplementary Fig. 2a). Insulin regulation of mTORC1 signaling was independent of Src activity (Supplementary Fig. 2b) while amino acid stimulation of mTORC1 was independent of Akt activity (Supplementary Fig. 2c). Activation of mTORC1 was further potentiated post amino acid stimulation (Fig. 2b), but this was attenuated by inhibition of Src in both *Tsc2*^−/−^ and *Tsc2*^*+/+*^ cells (Fig. 2c). mTORC1 localization at the lysosomal surface in response to amino acids was independent of TSC2 presence; however, mTORC1 translocation to lysosomes was decreased by PP2-mediated inhibition of Src in both *Tsc2*^*+/+*^ and *Tsc2*^*−/−*^ cells (Supplementary Fig. 2d, e) while, as expected, mTORC1 localization was independent of Akt activity. Together, these results establish that Src-dependent amino acid regulation of mTORC1 is independent of TSC2 signaling.

Next, we investigated the mechanism of mTORC1 activation by Src in response to amino acids. Rag GTPases are essential for the recruitment of mTORC1 at the lysosomal surface in response to amino acids. Previous reports have demonstrated that knockdown of the Rags releases mTORC1 from the lysosomal surface, thereby inactivating mTORC1 independently of amino acid availability^12,27^. We performed cell fractionation experiments and observed that Src is enriched with heavy membranes, similar to the lysosomal membrane proteins LAMP1 and LAMP2; gradient fractionation confirmed the presence of Src in the lysosome-enriched fraction (Fig. 2d, Supplementary Fig. 2f, g) with low amounts of Src in the cytosol. Confocal analysis showed that Src associates with lysosomes and RagC, independently of amino acid availability (Fig. 2e, f). We did not detect any obvious association of Src with other membrane-bound organelles such as the Golgi complex, mitochondria, peroxisomes, and the endoplasmic reticulum (Supplementary Fig. 2h). In addition, co-IP experiments showed that HA-GST-RagA/C interact with Src (Fig. 2g). Co-IP experiments confirmed that endogenous Src interacts with endogenous RagA and RagC (Fig. 2h) and that these interactions are independent of amino acid availability (Fig. 2i).

### Src promotes recruitment of mTORC1 at the lysosomal surface

**Fig. 3:**
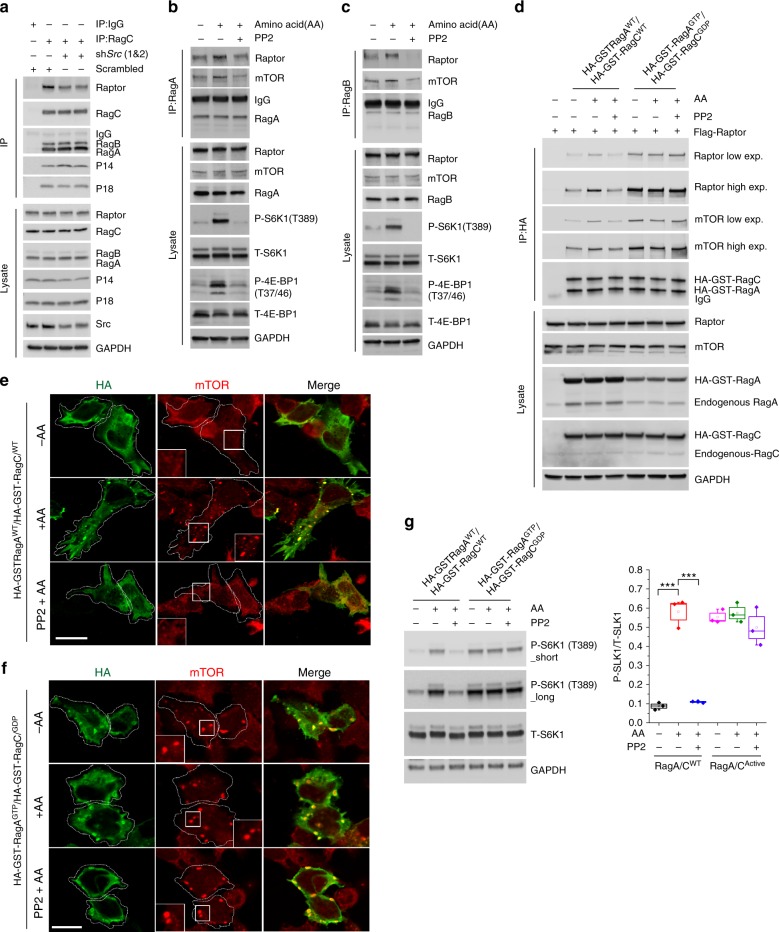
Src promotes mTORC1 recruitment to lysosomes. **a** HEK cells transiently transfected with scrambled shRNA or sh*Src* prior to coIP analyses. Lysates were used for immunoprecipitation by either Src or IgG antibody (negative control). **b**, **c** HEK293 cells were starved and treated with vehicle (DMSO) or PP2 (10 μM) for the last 2 h of starvation and then stimulated with amino acids (30 min). coIP analyses were performed to test interaction of mTOR and Raptor with RagA in **b** or RagB in **c**. Immunoblot analyses were used to measure the levels of the indicated proteins and phosphorylation states. **d** HEK293 cells stably expressing Flag-raptor, transiently transfected with HA-GST-RagA^WT^/C^WT^ or HA-GST-RagA^GTP^/C^GDP^, were starved and treated with vehicle (DMSO) or PP2 (10 μM) for the last 2 h of starvation and then stimulated with amino acids (30 min). coIP analyses were performed to test interaction of mTOR and Raptor with RagA/C. Immunoblot analyses were used to measure the levels of the indicated proteins. **e**, **f** HEK293 cells were transiently transfected with HA-GST-RagA^WT^/C^WT^ (**e**) or HA-GST-RagA^GTP^/C^GDP^ (**f**) and treated as in **a** prior to immunofluorescence labeling of HA (green) and endogenous mTOR (red). Representative cells are shown where punctate structures indicate lysosomal localization of mTOR. Magnified images are represented in white boxes. Bar indicates 40 μm. **g** HEK293 cells stably expressing HA-GST-RagA^WT^/C^WT^ or HA-GST-RagA^GTP^/C^GDP^ were treated as in **b**. Immunoblot analyses were used to measure the levels of the indicated proteins and phosphorylation states. Short and long indicate short and long exposure, respectively. Box plots represent SE of *n* = 3 independent experiments. ***p < 0.001. Statistical differences between groups were determined using ANOVA with Tukey’s post hoc test. GAPDH was used as a loading control in all immunoblot assays

We then investigated whether Src promotes mTORC1 recruitment at the lysosomal surface by regulating the GTP/GDP-bound state of the Rag GTPases in response to amino acids. We confirmed that Src regulates mTORC1 localization and activity at the lysosomal surface in response to amino acids in HEK293 cells (Supplementary Fig. 3a, b). Co-IP analysis in HEK293 cells showed that shRNA-mediated knockdown of *SRC* decreased the interaction of RagA with raptor, a component of mTORC1 complex^12^ (Fig. 3a). As a control, RagC interaction with RagA was not affected by knockdown of *SRC*. The specificity of the interaction between Rag and raptor was confirmed by IgG co-IP assay (Supplementary Fig. 3c). Co-IP analysis in HEK293 cells showed significantly stronger interactions of raptor and mTOR with the Rags in amino acid-stimulated cells compared to amino acid-depleted cells, which was suppressed by PP2 treatment (Fig. 3b, c and Supplementary Fig. 3d). mTORC1 activity was consistent with the status of the Rag–mTOR interaction (Fig. 3a, b). To determine whether Src regulates Rag–raptor interaction through the GTP/GDP loading status of the Rags, we used Rag nucleotide binding mutants that are either constitutively GTP-bound and therefore active (HA-GST-RagA^GTP^: Q66L)^27^ or that bind negligible amounts of nucleotides and are therefore inactive (HA-GST-RagC^GDP^: S75N)^27^. Co-IP experiments in cells stably expressing Flag-raptor showed that amino acid-mediated increases in both Rag–raptor and Rag–mTOR interactions were abolished by Src inhibition in cells expressing WT RagA/C, whereas both interactions became resistant to amino acid starvation or Src inhibition in cells expressing RagA^GTP^/RagC^GDP^ (Fig. 3d), thus suggesting that Src promotes Rag–mTOR interaction by regulating the GDP/GTP-loading status of the Rags.

Given that the Rag GTPases are responsible for the subcellular localization of mTORC1^12^ and Src regulates the Rag GTPases, we asked whether the observed effects of Src activity on mTOR localization depend on the Rags. Confocal analysis of cells expressing WT RagA/C showed that, upon amino acid deprivation, mTOR distributed in tiny puncta throughout the cytoplasm, whereas it localized to large vesicular structures in the perinuclear region in response to amino acid stimulation (Fig. 3e). In cells expressing constitutively active RagA/C (RagA^GTP^/RagC^GDP^), mTOR localized to the perinuclear vesicles independent of the availability of amino acids (Fig. 3f). PP2 was unable to alter the localization of mTOR in the presence of constitutively active RagA^GTP^/RagC^GDP^. Amino acid-mediated stimulation of mTORC1 activity was abolished by Src inhibition in cells expressing WT RagA/C, whereas mTORC1 activity became resistant to amino acid starvation or Src inhibition in cells expressing RagA^GTP^/RagC^GDP^ (Fig. 3g). Similar results were obtained in SHSY-5Y cells (Supplementary Fig. 3e). Together, these findings establish that Src regulates amino acid-mediated stimulation of mTORC1 activity via the Rag GTPases.

### Src disrupts GATOR1–Rag GTPase interaction

**Fig. 4:**
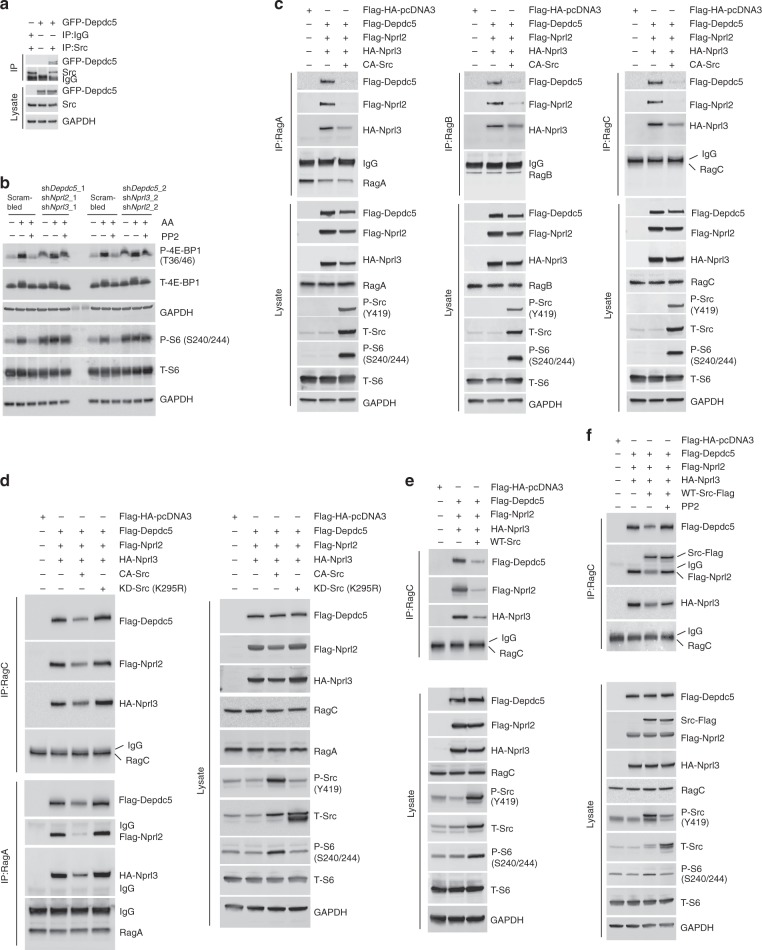
Src kinase disrupts the interaction of GATOR1 with the Rags. **a** Src kinase interacts with the GATOR1 complex. HEK293 cells, transiently transfected with GFP-Depdc5, were lysed and subjected to immunoprecipation with antibodies against Src or IgG (negative control) followed by immunoblotting for the indicated proteins. Lysates were probed with antibodies as indicated. **b** HEK cells, transiently transfected with scramble shRNA or shRNAs targeting *DEPDC5*, *NPRL2*, and *NPRL3* genes for 48 h prior to starvation and treatment with vehicle (DMSO) or PP2 for the last 2 h of starvation and then stimulated with amino acids (30 min). Immunoblot analyses were performed to measure the levels of the indicated proteins and phosphorylation states. **c** HEK293 cells, transiently transfected with the components of GATOR1 and constitutively active Src (CA-Src), were lysed and coIP analyses were performed to test interaction of GATOR1 with RagA, RagB, or RagC. **d** HEK293 cells, transiently transfected with GATOR1 components, CA-Src or kinase-dead Src (K295R), were lysed and coIP analyses were performed to test interaction of GATOR1 with RagA or RagC. **e** HEK293 cells, transiently transfected with the components of GATOR1 and wild-type Src (WT-Src), were lysed and subjected to RagC immunoprecipitation (IP) followed by immunoblotting for the indicated proteins. Lysates were probed with antibodies as indicated. **f** Inhibition of Src kinase promotes GATOR1–Rag interaction. HEK293 cells transfected as in **e** were treated with PP2 (5 μM, 24 h) prior to lysis, and then subjected to RagC immunoprecipitation (IP) followed by immunoblotting for the indicated proteins. GAPDH was used as a loading control in all immunoblot assays

A recent report identified GATOR1, a complex comprised of Depdc5, Nprl2, and Nprl3, as a negative regulator of mTORC1 that acts by increasing GTP hydrolysis of RagA and RagB^9^. Loss of GATOR1 function renders mTORC1 signaling insensitive to amino acid starvation^9^. Rag–GATOR1 interaction is decreased by amino acid stimulation^9^ and is independent of the availability of growth factors (Supplementary Fig. 4a). Since Src activation of mTORC1 is dependent on the GTP/GDP loading status of the Rags, we hypothesized that Src activation results in the disruption of GATOR1–Rag interaction. Co-IP assays in HEK293 cells transiently transfected with GFP-Depdc5 showed that Src interacts with GATOR1 (Fig. 4a). Consistent with a previous study^9^, we found that shRNA-mediated knockdown of GATOR1 (Supplementary Fig. 4b) decreased HEK cell size (Supplementary Fig. 4c) and blocked the inactivation of mTORC1 that is normally caused by amino acid deprivation (Supplementary Fig. 4d). To test whether Src regulation of mTORC1 activity is mediated through GATOR1, we knocked down GATOR1 by using shRNAs targeting *DEPDC5*, *NPRL2*, and *NPRL3* genes. While confirming that loss of GATOR1 makes mTORC1 insensitive to amino acid starvation, our results show that loss of GATOR1 makes mTORC1 activity resistant to Src inhibition (Fig. 4b), thus indicating that Src regulates mTORC1 via GATOR1. Next, we tested whether Src activates Rag–mTORC1 signaling by dissociating GATOR1 from Rag proteins. Expression of constitutively active Src (CA-Src) dramatically reduced GATOR1–Rag interaction (Fig. 4c); as a consequence, a strong activation of mTORC1 was observed. Importantly, expression of a kinase-dead Src construct (KD-Src/K295R-Src) did not alter Rag–GATOR1 interaction (Fig. 4d). Expression of WT-Src also decreased Rag–GATOR1 interaction (Fig. 4e), which was reversed by inhibition of Src with PP2 (Fig. 4f). Together, these results demonstrate that Src kinase regulates mTORC1 activity by controlling GATOR1–Rag interaction.

### Increased mTORC1 activity in cells with high Src activity

**Fig. 5:**
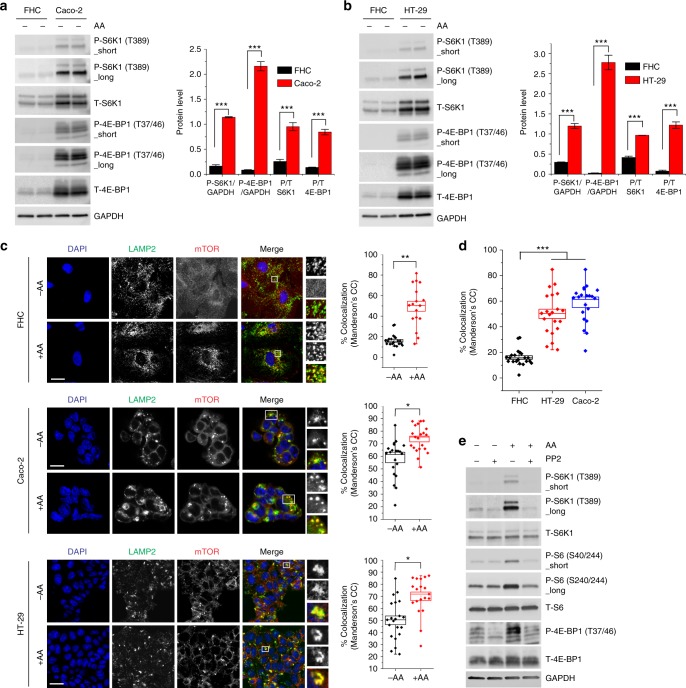
Increased mTORC1 activity in cells with elevated Src activity. **a** FHC and Caco-2 cells were amino acid starved (kept in dialyzed serum) for 4 h prior to lysis and immunoblot analysis to measure the levels of the indicated proteins and phosphorylation states. **b** FHC and HT-29 cells were treated as in **a** prior to immunoblot analysis to measure the levels of the indicated proteins and phosphorylation states. Quantified data in **a** and **b** represent means ± SEM of *n* = 2–3 independent experiments. **p < 0.001. **c** FHC, Caco-2 and HT-29 cells were starved as in **a** and then stimulated with amino acids for 30 min prior to immunofluorescence labeling of endogenous LAMP2 (green) and mTOR (red). Bars indicate 40 μm in FHC cells and 80 μm in Caco-2 and HT-29 cells. Representative cells are shown where yellow or orange pixels indicate co-localization in the merged images. Insets show selected fields that were magnified by a factor of 6. Quantified data represent co-localization (Mander’s coefficient) means of mTOR and LAMP2 in at least 15 cells for each condition. *p < 0.05 and **p < 0.01. **d** Extent of co-localization of mTOR and LAMP2 in FHC, Caco-2 and HT-29 cells under amino acid starvation. Box plots in **c** and **d** represent quantified data of co-localization (Mander’s coefficient) analysis in at least *n* = 30 cells/line. ***p < 0.001. **e** Caco-2 cells were amino acid starved (kept in dialyzed serum) for 4 h and treated with PP2 for the last 2 h prior to 30-min amino acid stimulation. Cells were lysed and immunoblot analyses were performed to measure the levels of the indicated proteins and phosphorylation states. Statistical differences between groups in **a**–**d** were determined using ANOVA with Tukey’s post hoc test

Given that Src positively regulates mTORC1 activity, we asked whether mTORC1 activity is higher in cells having endogenous activation of Src. To test this possibility, we investigated Src-mTORC1 signaling in HT-29 and Caco-2 colon cancer cells which have higher Src activity compared to normal colon cells^28–31^. We observed a significant increase in mTORC1 activity in both Caco-2 and HT-29 cells compared to the normal colon epithelial cell line FHC^28^ under amino acid starvation (Fig. 5a, b), similar to cells transfected with active Src constructs (Fig. 1g). Confocal microscopy experiments showed that amino acid stimulation increased mTOR translocation from the cytosol to lysosomes in FHC, Caco-2, and HT-29 cells (Fig. 5c); however, lysosomal localization of mTOR was significantly higher in Caco-2 and HT-29 cells compared to FHC cells under amino acid starvation (Fig. 5d). Interestingly, inhibition of Src abolished mTORC1 activity under both amino acid starvation and stimulation in Caco-2 cells (Fig. 5e), suggesting that increased activation of endogenous Src leads to higher mTORC1 activity as compared to cells with normal Src activity.

### Src regulates cell growth, TFEB localization, and autophagy

**Fig. 6:**
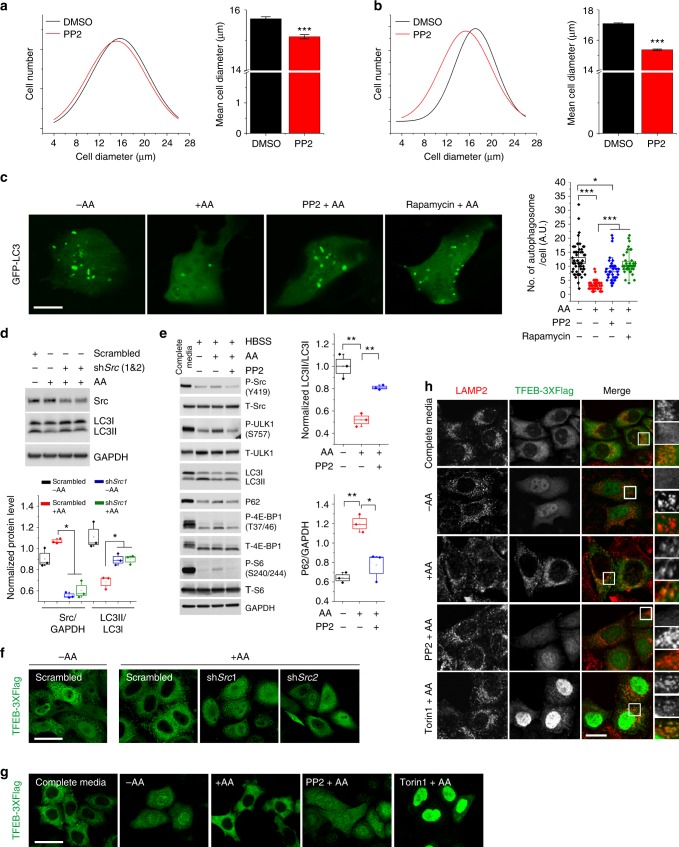
Src regulates cell growth, TFEB localization and autophagy. **a**, **b** The histogram shows the cell size of SH-SY5Y (**a**) and MEF (**b**) cells treated with DMSO or PP2 (5 μM for 24 h). The bar diagram represents the mean diameter of at least 10^4^ cells. Error bars represent values in ± SEM. ***p < 1 × 10^−6^. **c** SH-SY5Y cells expressing GFP-LC3 were starved and treated with vehicle (DMSO), PP2 (10 μM), or rapamycin (600 nM) for the last 2 h of starvation and then stimulated with amino acids (30 min). Box plots represent quantified data of lipidated-LC3 puncta from at least 35 cells. *p < 0.05 and ***p < 0.001. Bar indicates 20 μm. **d** Immunoblot analysis of SH-SY5Y cells transiently transfected with scrambled shRNA or sh*Src* prior to starvation and amino acid stimulation (30 min). **e** Immunoblot analysis of SH-SY5Y cells left in growth media or starved and treated with vehicle (DMSO) or PP2 (10 μM) for the last 2 h of starvation and then stimulated with amino acids (30 min). Box plots in **d** and **e** represent SE of *n* = 3 independent experiments. *p < 0.05 and **p < 0.01. **f** HeLa cells, stably transfected with Flag-TFEB, were transiently transfected with scrambled or sh*Src* and treated as in **d**. Cells were immunostained with Flag (green) for the analyses of cytosolic and nuclear localization of TFEB. Bar indicates 80 μm. **g** HeLa cells, stably transfected with Flag-TFEB, were treated as in **e**. Torin1 (300 nM for 2 h) was used as a control (mTOR inhibitor). Cells were immunostained with Flag (green) for the analyses of cytosolic and nuclear localization of TFEB. Bar indicates 40 μm. **h** HeLa cells, stably transfected with Flag-TFEB, were treated as in **e** prior to immunofluorescence labeling of LAMP2 (red) and Flag (green). Representative cells are shown where yellow or orange pixels indicate co-localization in the merged images. In all images, insets show selected fields that were magnified four times and their overlays. Bar indicates 30 μm. GAPDH was used as a loading control in all immunoblot assays. Statistical differences between groups in **a**–**e** were determined using ANOVA with Tukey’s post hoc test

Next, we investigated the role of Src in mTORC1 regulation of cell growth and autophagy. Prolonged cell exposure to PP2 significantly decreases the size of SH-SY5Y cells (Fig. 6a), MEFs (Fig. 6b), and HEK293 cells (Supplementary Fig. 5a), thus indicating that Src activity is necessary to sustain cell growth. Live imaging of SH-SY5Y cells transfected with GFP-LC3, a well-established probe to monitor autophagy induction^32,33^, showed that amino acid stimulation results in a decrease in LC3-positive autophagic vesicles compared to starved cells (Fig. 6c). Src inhibition, however, blocked amino acid-induced inhibition of autophagosome formation similar to the mTORC1 inhibitor rapamycin (Fig. 6c)^34^. Consistently, live imaging of HEK cells transfected with GFP-LC3 showed a significant reduction in LC3-positive autophagic vesicles in cells transfected with an shRNA targeting *SRC* (Supplementary Fig. 5b), thus indicating a critical role of Src in mTORC1 regulation of autophagy in response to amino acids. To test whether the observed increase in the number of autophagosomes was the result of an increase in the synthesis and not in the accumulation of the autophagosomes due to impairment of autophagic flux, we transfected cells with a tandem GFP-RFP-LC3 construct that emits a far-red fluorescent signal when autophagosomes fuse with lysosomes, and a yellow fluorescent signal when autophagosomes are unable to fuse with lysosomes^35,36^. A prevalence of red vesicles confirmed that LC3II-positive vesicles fused with lysosomes in the presence of PP2 (Supplementary Fig. 5c), indicating a functionally active autophagic system. Consistent with these results, immunoblot assays showed an increase in the amount of LC3II and a concomitant decrease in P62 level upon knockdown of *SRC* (Supplementary Fig. 5d). In addition, amino acid-mediated decrease in the amounts of LC3II was significantly reversed upon knockdown of *SRC* (Fig. 6d). Consistently, a reduction in the amounts of LC3II and a concomitant increase in mTORC1 activity were observed upon amino acid stimulation and reversed by Src inhibition (Fig. 6e). Similar results were obtained in HEK293 cells (Supplementary Fig. 5e).

mTORC1 regulates the expression of autophagy and lysosomal genes by controlling the activity of the transcription factor EB (TFEB), a master regulator of autophagy and lysosomal genes^26,37–39^. TFEB phosphorylation by mTORC1 promotes TFEB retention in the cytosol, thus hampering its positive regulation of transcription^40^. Amino acid signaling to mTORC1 correlates with TFEB subcellular localization^41^. We tested whether Src regulation of mTORC1 activity modulates the localization of TFEB in response to amino acids. Confocal microscopy showed that amino acid starvation increases nuclear translocation of TFEB, whereas amino acid stimulation blocked nuclear translocation of TFEB (Fig. 6g), as previously reported^42^. ShRNA-mediated knockdown of *SRC* promoted TFEB nuclear translocation in the presence of amino acids (Supplementary Fig. 5f and Fig. 6f). Consistently, pharmacological inhibition of Src showed a significant increase in nuclear TFEB similar to Torin1 (Fig. 6g), a catalytic inhibitor of mTOR^43^. TFEB is known to localize to lysosomes in an amino acid-dependent manner via recruitment by active Rags^42^. In cells stimulated with amino acids, TFEB showed an increased association with lysosomes (Fig. 6h). Strikingly, pretreatment with PP2—which as shown above causes inhibition of both Rag and mTORC1 activity—resulted in a substantial translocation of TFEB to the nucleus, with no detectable association of TFEB with lysosomes. In contrast, pretreatment with Torin1 resulted in a dual localization of TFEB to the nucleus and to lysosomes, consistent with the notion that Torin1 inhibits mTORC1 activity but not amino acid-dependent activation of the Rags. Collectively, these results demonstrate that Src controls the activity of the Rag GTPases and mTORC1 and the subcellular localization of TFEB in response to amino acids, thereby regulating autophagy and cell growth.

## Discussion

**Fig. 7:**
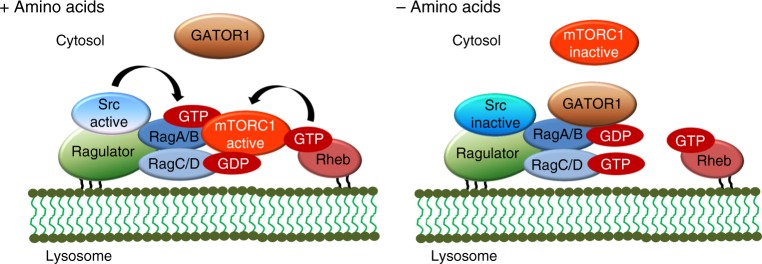
Schematic diagram for Src-dependent regulation of mTORC1. The model shows that Src kinase regulates amino acid-mediated mTORC1 activity by controlling GATOR1–Rag interaction

Results from this study define Src as an essential upstream positive regulator of amino acid-induced mTORC1 signaling (Fig. 7). Src regulates localization and activation of mTORC1 upon amino acid stimulation, and Src activity per se is sufficient for the activation of mTORC1, irrespective of amino acid availability. Our work also leads to a number of novel questions which will be interesting to explore in future studies. First, what is the upstream mechanism that activates Src in response to amino acids? Sestrin2 and CASTOR1 were characterized as sensors for cytosolic leucine and arginine, respectively^44–46^. Upon amino acid binding to sestrin2 or CASTOR1, GATOR2 dissociates from them and initiates GTP-loading of RagA/B via inhibition of GATOR1^14,15^. It is possible that GATOR2 transfers the amino acid signal to Src, which then initiates GTP-loading of RagA/B by dissociating Rag–GATOR1 interaction. GATOR2, once released from sestrin2 or CASTOR1, might interact with Src and lead to a conformational change that activates Src thus enabling dissociation of GATOR1 from the Rags. Future studies that investigate the molecular association between GATOR2 and Src could establish whether this mechanism funnels so far unidentified regulatory signals to GATOR1 so as to modulate amino acid sensing by mTORC1. Second, how does activation of Src dissociate GATOR1 from the Rags? As the kinase activity of Src is involved in determining GATOR1 interaction with Rags, a possibility is that Src, directly or indirectly (via another kinase) phosphorylates some component(s) of GATOR1 and this event could be critical for Rag–GATOR1 complex formation. Alternatively, amino acid-mediated activation of Src could lead to the disruption of the GATOR1 complex itself.

The characterization of the mechanism by which Src dissociates GATOR1 from the Rags also warrants further investigation. Sustained activation of mTORC1 is essential for cells to grow to their optimal size^12^, and our results indicate that Src regulates mTORC1-dependent cell growth as well as autophagy. This regulatory pathway might be disrupted in several human pathological conditions such as cancer and various metabolic disorders with mTORC1 involvement. Taken together, our study leads to the unexpected finding that Src is a crucial upstream regulator of mTORC1 that can sustain mTORC1 activity and promote cell survival under nutrient deprivation conditions, which might have a therapeutic potential in mTOR-associated pathologies.

## Methods

### Materials

Reagents were obtained from the following sources: DMEM/F12 (1:1) from life technologies (11320033); DMEM from hyclone (SH30081); dialyzed fetal bovine serum (dFBS) from GE Healthcare Life Sciences (SH30079); amino acids from Sigma Aldrich (A6282); Rapamycin from Sigma Aldrich (R0395); PP2 from Calbiochem (529573) and Dasatinib from LC Laboratories (D-3307). MK2206 was from Selleckchem and insulin was obtained from Sigma Aldrich.

### Cell culture

SHSY-5Y cells (ATCC® CRL2266™) were grown in DMEM-F12 (1:1, Gibco) supplemented with 10% heat inactivated fetal bovine serum (FBS, Atlanta Biologicals), 2 mM l-glutamine, 100 U/ml penicillin and 100 mg/ml streptomycin (Gibco) (referred as nutrient-rich media in manuscript). HEK293T, HeLa, and MEF cells were cultured in DMEM supplemented with 10% heat inactivated FBS, 2 mM l-glutamine, 100 U/ml penicillin, and 100 mg/ml streptomycin. FHC, Caco-2, and HT-29 cells were purchased from ATCC and cultured according to the manufacturer’s protocol. HEK293T cells stably expressing Flag-raptor were a generous gift from Kartik Venkatachalam (UT Health Science Center). WT MEF cells were generated and cultured in our laboratory. *Tsc2*^*−/−*^ MEFs were kindly provided by Dr. Krymskaya (University of Pennsylvania).

### Plasmids

For the generation of WT-Src-Flag construct, Src cDNA was amplified from pcDNA3 c-SRC (WT) (Addgene #42202) kindly provided by Robert Lefkowitz and cloned into XbaI and HindIII sites of p3XFLAG-CMV-14 (Sigma). SrcΔSH3-Flag and SrcΔSH2-Flag constructs were generated using site-directed mutagenesis (Promega) with appropriate sets of primers. In brief, for the SrcΔSH2-Flag, we first inserted 2 PacI restrictions sites flanking the SH2 domain by using the following two primers pairs: 5prime_for: 5’-CCAGGCTGAGGAGTTTAATTAAGGCAAGATCACCA-3’, 5prime_rev: 5’ TGGTGATCTTGCCTTAATTAAACTCCTCAGCCTGG-3’, 3prime_for: 5’-ACCACCGTGTGCCCCATTAATTAACCGCAGACTCAGGGC-3’, and 3prime_rev: 5’- GCCCTGAGTCTGCGGTTAATTAATGGGGCACACGGTGGT-3’. After SH2 deletion, the construct was codon optimized using the following primers, 5’- ATCCAGGCTGAGGAGTGGTATTTTCCGCAGACTCAGGGC-3’ and 5’- GCCCTGAGTCTGCGGAAAATACCACTCCTCAGCCTGGAT-3. For the SrcΔSH3-Flag, we inserted a ApaI site at the end of the SH2 domain by using the following primers 5’- ATCCAGGCTGAGGGCCCGTATTTTGGCAAG-3’ and 5’- CTTGCCAAAATACGGGCCCTCAGCCTGGAT-3’. The construct was then digested with ApaI and re-ligated without the SH3 domain. For the generation of CA-Src (Y530F-Src) we used the following primers: 5’-GTCCACCGAGCCCCAGTTCCAGCCCGGGGA-3’ and 5’-TCCCCGGGCTGGAACTGGGGCTCGGTGGAC-3’. The fllowing constructs were obtained from Addgene: Flag-HA-pcDNA3 (# 10792), KD-Src (K295R-Src) (#13659), HA-GST-RagA (#19298), HA-GST-RagB (#19301), HA-GST-RagC (#19304), HA-GST-RagA^GTP^:Q66L (#19300), HA-GST-RagC^GDP^:S75L (#19305), Flag-Depdc5 (#46350), GFP-Depdc5 (#46380), Flag-Nprl2 (#46333), and HA-Nprl3 (#46330). All constructs were sequence varified. GFP-LC3 and GFP-RFP-LC3 constructs were kindly provided by Ross Poche (Baylor College of Medicine).

### Starvation protocol

For amino acid stimulation studies, sub-confluent cells were serum-starved for 16 h followed by amino acid starvation for 4 h prior to stimulation with a 10× mixture of total amino acids for 30 min, unless otherwise indicated. The final concentration of amino acids used to stimulate the cells was 2×. The 10× mixture of total amino acids was prepared from individual powders of amino acids^9,12^. For amino acid deprivation studies, complete growth medium was replaced by dialyzed serum for 4 h. The recipe of the 10× mixture of total amino acids is provided in Supplementary Table 1. Chemical treatments were performed with indicated drugs for the last 2 h of amino acid starvation.

All the dry/powder amino acids were added to 500 ml of dH_2_O. Another 476 ml of dH_2_O was added to make final volume 1 l. Solution was mixed thoroughly by shaking and heating (mild) on a stir plate followed by filter sterilization using 0.2 micron filtration units.

### Cell lysis and immunoblotting

After the end of treatment, cells were rinsed once with ice-cold PBS and harvested, and lysed using RIPA buffer (50 mM Tris-HCl, ph 7.4, 1% NP40, 0.5% Na-deoxycholate, 0.1% SDS, 150 mM NaCl, 2 mM EDTA, and 50 mM NaF) including a cocktail of protease (Roche, Basel, Switzerland) and phosphatase (MilliporeSigma, Billerica, MA) inhibitors. Protein concentration was measured with the bicinchoninic acid (BCA) protein assay kit (Pierce, Rockford, IL), using BSA as standard. Lysates were separated via SDS-PAGE and then transferred to polyvinyldifluoride (PVDF) membranes. Blots were incubated in blocking buffer (5%, w/v, dried skimmed milk in Tris-buffered saline, pH 7.4, and 0.2% Tween 20, TBST) followed by overnight incubation with appropriate antibodies diluted in blocking buffer. Blots were then exposed to the IRDye® secondary antibodies (LI-COR, Lincoln, NE) or HRP-conjugated antibodies diluted in TBST or blocking buffer, respectively, for 75 min at room temperature and washed again. Blots were detected using appropriate image-developer. Uncropped scans of all blots are provided in Supplementary Fig. 6.

### Immunofluorescence

Cells were grown on glass coverslips in 24-well plates. After the end of the treatment, cells were rinsed once with PBS and fixed with 1× PBS-4% paraformaldehyde at room temperature (RT) for 15 min. Cells were rinsed three times with PBS, permeabilized with 0.1% Triton X-100 in 1X PBS for 5 min, rinsed twice with PBS, and blocked with blocking reagent (0.1% saponin, 8% goat serum in PBS) for 1 h at RT. After one rinse with PBS, cells were incubated with primary antibody in blocking reagent overnight at 4 °C in a humidity chamber. After three PBS washes, coverslips were incubated with labeled secondary antibodies for 1 h at RT in the dark. After four PBS washes, coverslips were mounted with vectashield containing DAPI (H-1200) prior to microscopy. Images were acquired through × 63 or × 40 oil immersion objectives with either a Zeiss Axiotome fluorescence microscope with Apotome feature engaged or a Zeiss 710 confocal laser microscope (Zeiss, Oberkochen, Germany). Representative images are shown in all figures at the same exposure and magnification. Antibody details are provided in Supplementary Table 2.

### Immunoprecipitation

For the immunoprecipitation analyses regulator components, cells were rinsed once with ice-cold PBS and lysed with ice-cold Chaps lysis buffer (0.3% Chaps, 10 mM β-glycerol phosphate, 10 mM pyrophosphate, 40 mM Hepes pH 7.4, 2.5 mM MgCl_2_, and 1 tablet of EDTA-free protease inhibitor (Roche) per 25 ml). For all other immunoprecipitation assays, Cells were rinsed once with ice-cold PBS and lysed in ice-cold lysis buffer (40 mM HEPES [pH 7.4], 2 mM EDTA, 10 mM pyrophosphate, 10 mM glycerophosphate, and 1% Triton X-100, and one tablet of EDTA-free protease inhibitors (Roche) per 25 ml). The soluble fractions of cell lysates were isolated by centrifugation at 19,000 × *g* for 15 min at 4 °C. Protein concentration was measured with the BCA protein assay kit (Pierce, Rockford, IL). Primary antibodies were added to the lysates and incubated with rotation overnight at 4 °C. 60 μl of a 50% slurry of protein G-agarose beads (Roche, Basel, Switzerland) was added and incubation continued for 4 h at 4 °C. Immunoprecipitates were washed three times (7 min each at 4 °C) with lysis buffer containing 150 mM NaCl. Immunoprecipitated proteins were denatured by the addition of 20–30 μl of sample buffer (including 2-mercaptoethanol) and boiling for 5 min, resolved by 4–20% SDS-PAGE (Initrogen, Carlsbad, CA), and analyzed by immunoblotting. For co-transfection experiments, 1.5 × 10^6^ cells were plated in 10 cm culture dishes. Twenty hours later, cells were transfected with the appropriate expression plasmids indicated in the figures in equal amounts. The total amount of plasmid DNA in each transfection was normalized to 5 μg with empty vector. Thirty-six hours after transfection, cells were lysed as described above.

### Cytosolic/light membrane and heavy membrane fractions

SH-SY5Y cells were grown in two 15-cm dishes per condition. Cells (90–95% confluent) were rinsed with ice-cold PBS, scraped into ice-cold PBS, pelleted by centrifugation at 1000 × *g* for 2 min at 4 °C. Supernatants were discarded and pellets were resuspended in 350 μl cold hypotonic lysis buffer (10 mM HEPES, pH 7.2, 10 mM KCl, 2 mM MgCl2, 20 mM NaF, 100 μM sodium orthovanadate, 250 mM sucrose with freshly added protease, and phosphatase inhibitors). Cells were mixed thoroughly by vortexing. Cells were mechanically lysed by drawing five times through a 23G needle and centrifuged at 500 × *g* for 10 min at 4 °C, yielding a post-nuclear supernatant (PNS). PNS was used for the fractionation of light membrane/cytosolic and heavy membrane fractions. The PNS was centrifuged at 20,000 × *g* for 2 h to separate the soluble supernatant (cytosolic/light membrane fraction) from the insoluble pellet (heavy membrane fraction). The pellet was lysed in RIPA buffer (50 mM Tris-HCl, ph 7.4, 1% NP40, 0.5% Na-deoxycholate, 0.1% SDS, 150 mM NaCl, 2 mM EDTA, and 50 mM NaF) including a cocktail of protease (Roche) and phosphatase (MilliporeSigma) inhibitors. Protein concentration was measured with the BCA protein assay kit (Promega) using BSA as standard. Proteins were denatured by the addition of 20–30 μl of sample buffer (including 2-mercaptoethanol) and boiling for 10 min, resolved by 4–20% SDS-PAGE (Invitrogen), and analyzed by immunoblotting.

### Separation of lysosome-enriched fraction

Lysosomes were enriched from SH-SY5Y cells from three near-confluent 15-cm dishes. Briefly, cells where washed with ice-cold PBS, pelleted at 500 × *g* for 10 min in assay buffer (0.25 M sucrose, pH 7.2) and homogenized using a Dounce homogenizer. The homogenate was centrifuged at 800 × *g* for 10 min, the supernatant was collected separately, and the pellet was rinsed with assay buffer followed by another centrifugation at 800 × *g* for 10 min. The supernatants from both centrifugations were combined, mixed thoroughly with assay buffer and Percoll (20% final concentration) by vortexing. Samples were centrifuged at 36,000 × *g* for 30 min using SW 40 Ti rotor (Beckman). A total of 10 fractions were collected. Fractions were diluted in 5–10 volumes of assay buffer and centrifuged at 36,000 × *g* for 15 min. Pellets were then resuspended in 200 μl of RIPA buffer. These samples were then analyzed by immunoblotting assay as described earlier.

### Cell size determination

SH-SY5Y, MEF, and HEK293T cells were cultured as described earlier. Cells, 70–80% confluent, were then treated with DMSO or 5 μM PP2 for 24 h. After the treatment, cells were washed with 1× PBS twice, trypsinized and resuspended to a concentration of 1 × 10^5^ cells per ml in 1× PBS. Cell number and size of each condition was measured using the size histogram function of the Cellometer Auto 1000 (Nexcelom Bioscience, USA). Line-plot profile was created from binned-histogram generated from a total of at least 1 × 10^4^ cells per condition using Origin Pro software (OriginLab Corporation, Northhampton, MA).

### shRNA knockdown

shRNA-mediated knockdowns were performed using jetPRIME transfection reagent (Polyplus, Illkirch France). Cells were transfected according to the manufacturer’s protocol and processed for the given experiments at 70–72 h post-transfection. SH-SY5Y cells transfected with shRNA-Src or scrambled (ORIGENE, Rockville, MD) were maintained in DMEM-F12/10% FBS. For the amino acid-stimulation experiments, 50 h after transfection cells were starved of serum (16 h) and amino acids (last 4 h) followed by stimulation with amino acids (30 min). shRNA-mediated knockdowns of GATOR1 genes were performed in HEK cells using jetPRIME transfection reagent (Polyplus, Illkirch France). Cells were transfected according to the manufacturer’s protocol and processed for the given experiments 48 h post-transfection. For the amino acid-stimulation experiments, 48 h after transfection the cells were starved of serum (16 h) and amino acids (last 4 h) followed by 2 h of PP2 treatment (last 2 h of amino acid starvation) prior to stimulation with amino acids (30 min).

The following Src shRNAs were used:

shSrc1: 5’-ATGACCTTATGTGCCAGTGCTGGCGGAAG-3’;

shSrc2: 5’-CAGATCGCTTCAGGCATGGCCTATGTGGA-3’;

shSrc3: 5’-CTGGAGGCAATCAAGCAGACATAGAAGAG-3’.

Scrambled: Non-effective 29-mer scrambled shRNA cassette (OriGene, TR30015)

shRNAs targeting components of GATOR1 complex were purchased from Cell-Based Assay Screening Service (C-BASS) in Baylor College of Medicine. The following shRNAs were used:

Depdc5_1: 5’-AGGTCAAGTCTCTTAAGGA-3’

Depdc5_2: 5’-AGGAGTGTGCACAGATGTA-3’

Nprl2_1: 5’-CCTGTCTTTACCAAAGACA-3’

Nprl2_2: 5’-CGGGATGTGTTCCAGCTAT-3’

Nprl3_1: 5’-GTGATAAACTGTCTGCATA-3’

Nprl3_2: 5’-ATGTTATTCTGGCAACAAT-3’

### Quantitative real-time PCR

Total RNA was extracted from HEK cells transiently transfected with scramble shRNA or shRNAs targeting *DEPDC5*, *NPRL2* and *NPRL3* genes for 48 h. RNA was isolated using the RNEasy kit (Qiagen) according to the manufacturer’s instructions. RNA quantification was performed using the Nano-Drop 8000 (Thermo Fischer) followed by cDNA synthesis using QuantiTect Reverse Transcription kit (Qiagen). Quantitative real-time PCR was performed by using iQ SYBR Green Supermix (Bio-rad, cat# 172-5124) on the CFX96 Touch Real-Time Detection System (Bio-Rad Laboratories). Analyses were conducted using CFX manager software (Bio-Rad) and the threshold cycle (CT) was extracted from the PCR amplification plot. The change in messenger RNA levels was expressed as fold change. The primers used for qRT-PCR are indicated below.

NPRL2_Forward: TGGGACCCAAGATCACCTAT

NPRL2_Reverse: GCTTGTTCTGCAGCTCTGG

NPRL3_Forward: GGGCAGATCTCCAAAACAGA

NPRL3_Reverse: ACGGGACAGGTTATGCAGAC

DEPDC5_Forward: TAACCCACAGTTCACCAGCC

DEPDC5_Reverse: GGGGATATGTGGCGACTAAA

### Statistics

Bar diagrams are reported as mean ± SEM and Box plots are reported as SE,unless otherwise specified. Statistical differences between groups were determined using ANOVA with Tukey’s post hoc test. Statistical analysis was performed in Origin Pro (OriginLab Corporation, Northhampton, MA) with a significance level of *p < 0.05, **p < 0.01, and ***p < 0.001.

## Electronic supplementary material

Supplementary Information

## Data Availability

The data that support the findings of this study are available from the authors on reasonable request.
