# Supplementary material for: Src regulates amino acid-mediated mTORC1 activation by disrupting GATOR1-Rag GTPase interaction
Source: Nat Commun. 2018 Oct 19;9:4351. doi: 10.1038/s41467-018-06844-4 (PMC6195609; doi:10.1038/s41467-018-06844-4)
Supplement: Supplementary file 1 — Supplementary Information [file 41467_2018_6844_MOESM1_ESM.pdf]

Supplemental Information

Src regulates amino acid-mediated mTORC1 activation by disrupting GATOR1-Rag GTPase interaction

Pal. et al.

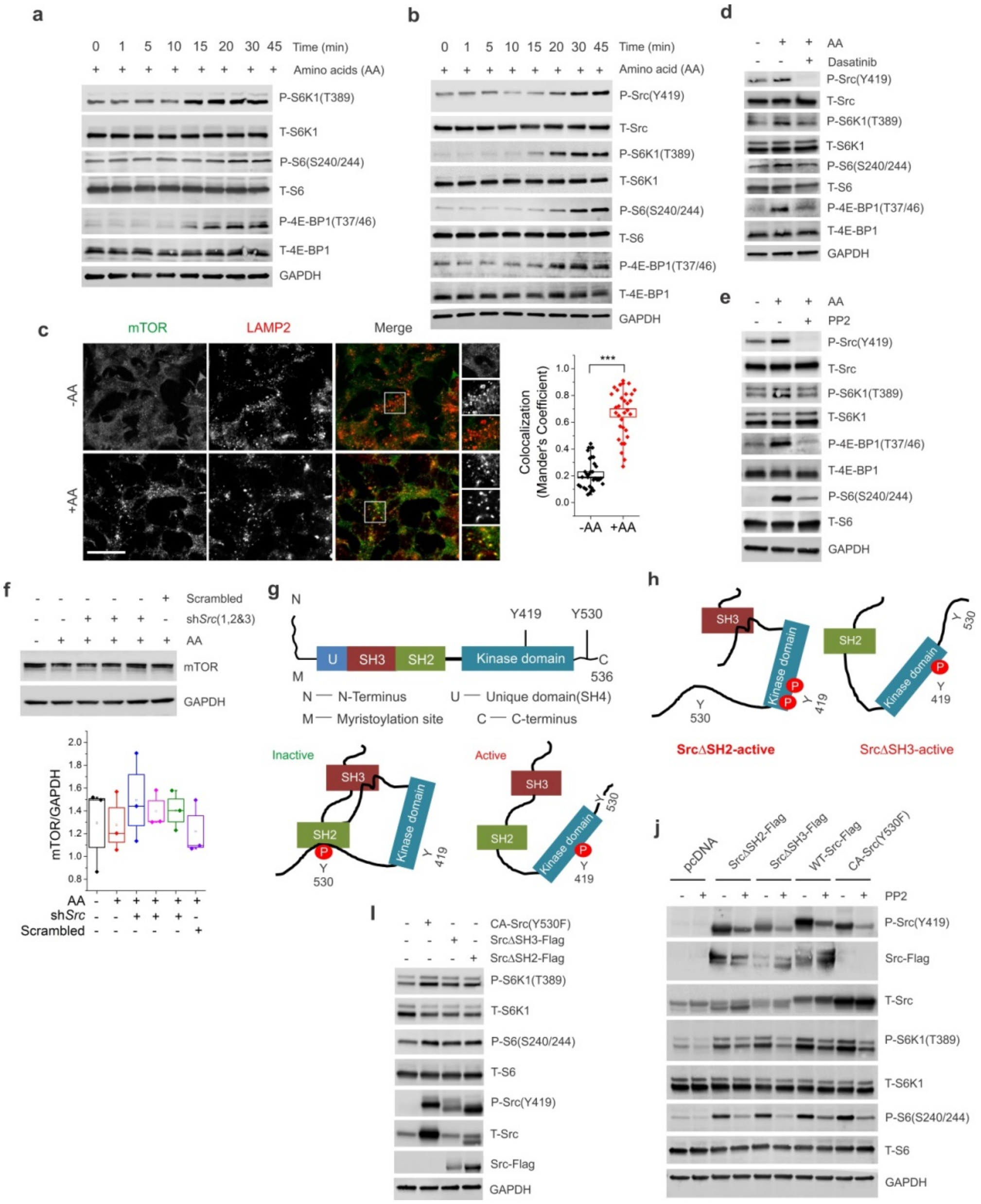

Supplementary Figure 1 (**a**,**b**) SH-SY5Y and MEF cells were starved and then subjected to time-course amino acid stimulation. Immunoblots of lysates were probed with antibodies as indicated. (**c**) SH-SY5Y cells were treated as in (**a**) but with a single 30 min-amino acid stimulation prior to immunofluorescence labeling of endogenous LAMP2 (red) and mTOR (green). Representative cells are shown where yellow or orange pixels indicate co-localization in the merged images. In all images, insets show selected fields that were magnified by a factor of 4. Bar indicates 60 μm. Box plots represent quantified data of percent colocalization (Mander's coefficient) of mTOR and LAMP2 in at least n = 30 cells for each condition. ***p<0.001. Statistical differences between groups were determined using ANOVA with Tukey's post-hoc test. (**d**,**e**) SH-SY5Y cells starved and treated with vehicle (DMSO) or Dasatinib (600 nM) (**d**) or PP2 (**e**) for the last 2 h of starvation and then stimulated with amino acids (30 min). Immunoblot of lysates were used to measure the levels of the indicated proteins and phosphorylation states. (**f**) SH-SY5Y cells, treated with shRNAs targeting the mRNA for the Src kinase followed by starvation and amino acid stimulation for 30 min. Immunoblot analyses were used to measure the levels of the indicated proteins. The box plots represent SE of n = 3 independent experiments. (**g**) Model diagram shows the structure of Src kinase and conformations of Src kinase in active-and-inactive states. When phosphorylated, Src tyrosine 530 (pY530) binds intramolecularly to the SH2 domain, which keeps the kinase in a closed and inactive condition. In the active state, conformation changes to dephosphorylation at Y530 and phosphorylation at Y419. (**h**) Models show conformation of Src kinase in active states. Deletion of SH2 or SH3 domain leads to an open conformation of Src that induces dephosphorylation at Y530 and phosphorylation at Y419. (**i**) SH-SY5Y cells were transiently transfected with Y530F-Src, SrcΔSH3 or SrcΔSH2. Immunoblot analyses were used to measure the levels of the indicated proteins and phosphorylation states. (**j**) SH-SY5Y cells, transiently transfected with the indicated Src constructs, were treated with vehicle (DMSO) or PP2 (10 μM). Immunoblot analyses were used to measure the levels of the indicated proteins and phosphorylation states. GAPDH was used as a loading control in all immunoblot assays.

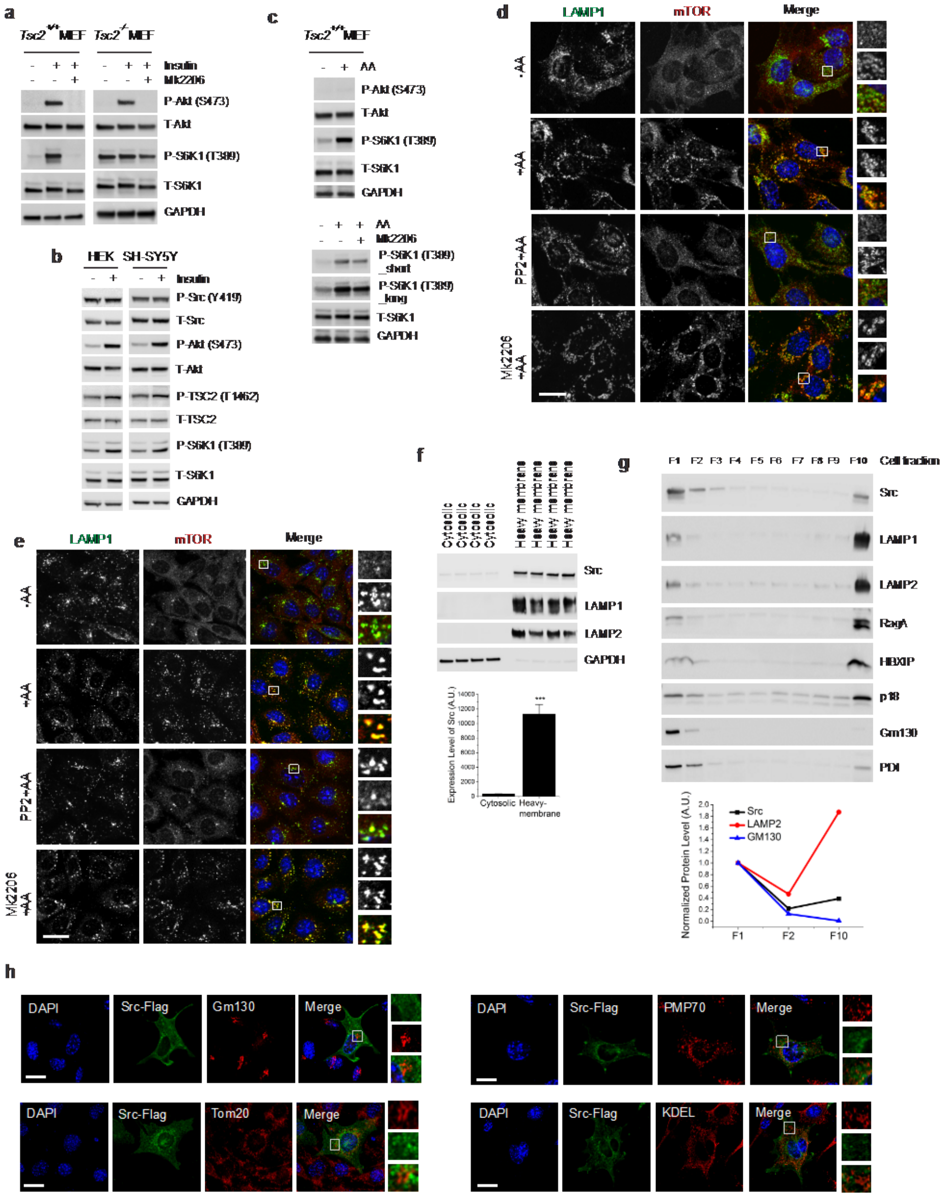

Supplementary Figure 2 (**a**) *Tsc2*$^{+/+}$ and *Tsc2*$^{-/-}$ MEF cells were starved of serum (16 hr) and treated with DMSO or MK2206 prior to insulin stimulation (1 µM, 15 min). Immunoblot analyses were performed to assess the expression of indicated proteins. (**b**) HEK and SH-SY5Y cells were starved of serum (16 hr) prior to insulin stimulation (1 µM, 15 min). Immunoblot analyses were performed to assess the expression of indicated proteins. (**c**) MEF cells were starved and then stimulated with amino acids for 30 min (Top) or treated with DMSO or MK2206 prior to amino acid stimulation. Immunoblots of lysates were probed with antibodies as indicated. (**d**,**e**) *Tsc2*$^{+/+}$ (**d**) and *Tsc2*$^{-/-}$ (**e**) MEFs were starved and treated with vehicle (DMSO) or PP2 for the last 2 h of starvation and then stimulated with amino acids (30 min) prior to immunofluorescence labeling of endogenous LAMP1 (green) and mTOR (red). Representative cells are shown where yellow or orange pixels indicate colocalization in the merged images. In all images, insets show selected fields that were magnified by a factor of 4. Bar indicates 30 µm. (**f**) SH-SY5Y cells were lysed and separated into cytosolic/light membrane and heavy membrane fractions. Immunoblot analyses were used to measure the levels of the indicated proteins. The bar diagram represents average densitometry values from four replicates with an error of ±SEM. ***p<0.001. Statistical differences between groups were determined using ANOVA with Tukey's post-hoc test. (**g**) SH-SY5Y cells were lysed and plasma membrane-fraction was discarded. Sub-cellular fractionation analysis was performed with rest of the cell lysates. Fraction 1&2 (F1&F2) contain whole organelles population, whereas Fraction 10 (F10) represents the lysosome-enriched fraction. LAMP1, LAMP2, RagA, HBXIP and p18 are lysosomal-associated proteins while GM130 and PDI are predominantly present in the Golgi and endoplasmic reticulum, respectively. Line-plot profile shows GM130 and PDI were decreased in F10 compared to F2 while levels of LAMP1 and Src kinase were increased in F10 compared to F2. (**h**) MEF cells were transfected to express Src prior to immunofluorescence labeling of GM130 (Golgi marker, red) and Src (green), Tom20 (mitochondrial marker, red) and Src (green), PMP70 (peroxisome, red) and Src (green) and KDEL (endoplasmic reticulum marker, red) and Src (green). Representative cells are shown in the merged images. In all images, insets show selected fields that were magnified by a factor of 4. Bar indicates 40 µm. GAPDH was used as a loading control in all immunoblot assays.

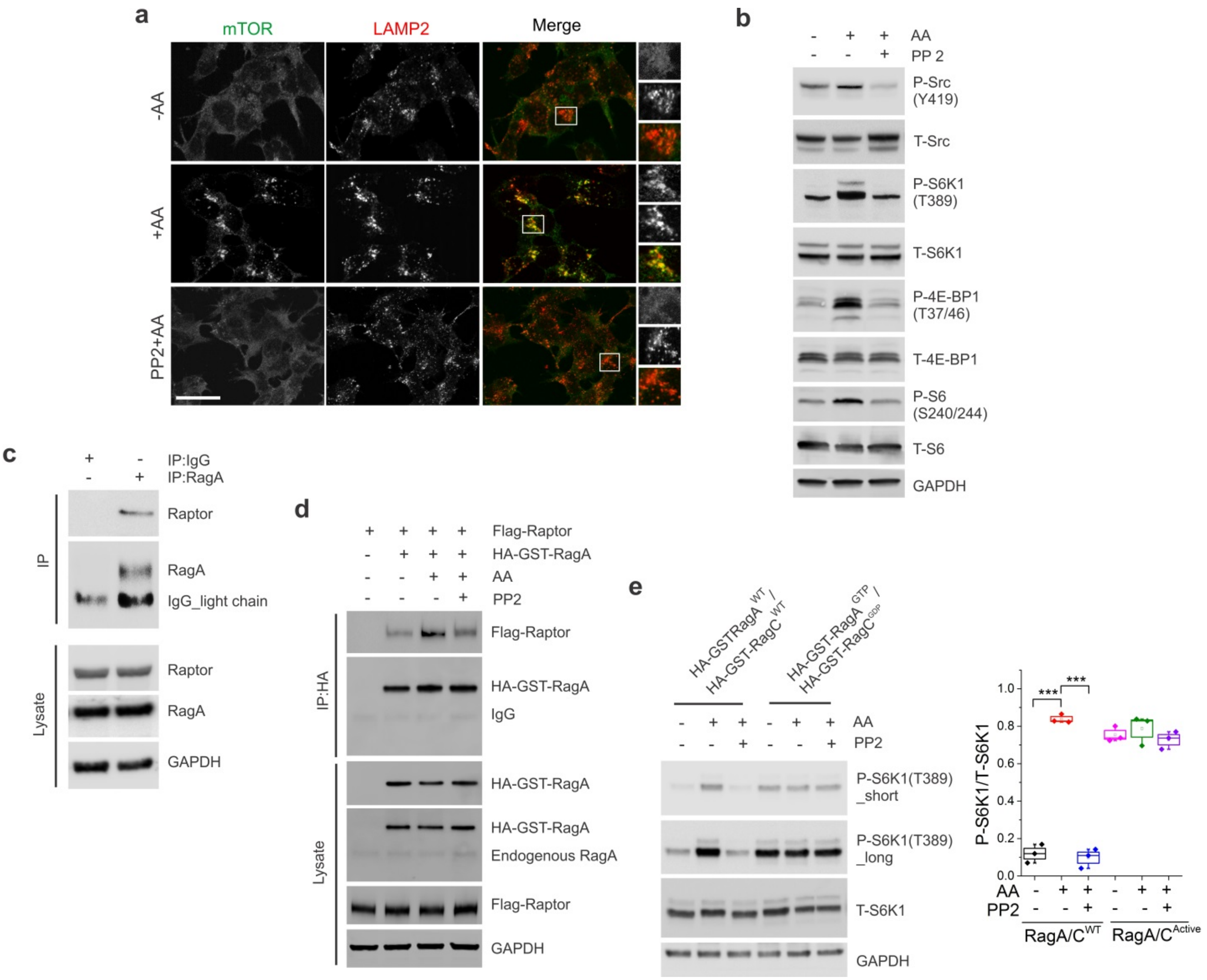

Supplementary Figure 3 (**a**) HEK293 cells were starved and treated with vehicle (DMSO) or PP2 for the last 2 h of starvation and then stimulated with amino acids (30 min) prior to immunofluorescent labeling of endogenous LAMP1 (green) and mTOR (red). Representative cells are shown where yellow or orange pixels indicate colocalization in the merged images. In all images, insets show selected fields that were magnified by a factor of three and their overlays. Bar indicates 60 μm. (**b**) HEK293 cells were treated as in (**a**) prior to immunoblot analyses to measure the levels of the indicated proteins and phosphorylation states. (**c**) HEK293 cells were lysed and subjected to IgG or RagA immunoprecipitation (IP) followed by immunoblotting for the indicated proteins. Lysates were probed with antibodies as indicated. (**d**) HEK293 cells stably expressing Flag-raptor, transiently transfected with HA-GST-RagA^{WT}, were starved and treated with vehicle (DMSO) or PP2 (10 μM) for the last 2 h of starvation and then stimulated with amino acids (30 min). CoIP analyses were performed to test interaction of Raptor with RagA. Immunoblot analyses were used to measure the levels of the indicated proteins. (**e**) SH-SY5Y cells stably expressing HA-GST-RagA^{WT}/C^{WT} or HA-GST-RagA^{GTP}/C^{GDP} were treated as in (**d**). Immunoblot analyses were used to measure the levels of the indicated proteins and phosphorylation states. The box plots represent SE of n = 3 independent experiments. ***p<0.001. Statistical differences between groups were determined using ANOVA with Tukey's post-hoc test. GAPDH was used as a loading control in all immunoblot assays.

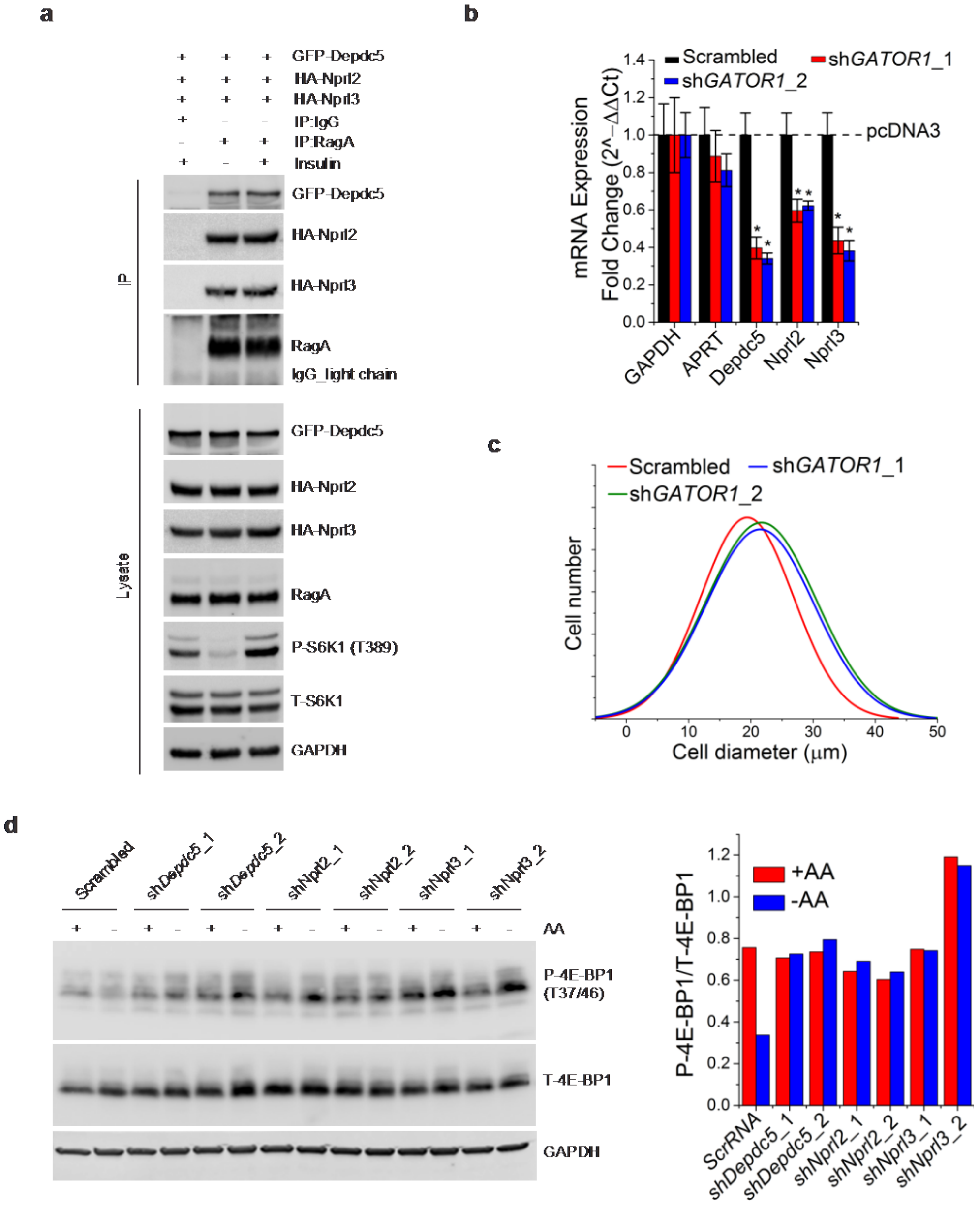

Supplementary Figure 4 (**a**) HEK293 cells, transiently transfected with GATOR1 components (Depdc5, Nprl2 and Nprl3), were starved of serum (16 hr) prior to insulin stimulation (1 µM, 15 min). Cells were then lysed and subjected to RagA immunoprecipitation (IP) followed by immunoblotting for the indicated proteins. Lysates were probed with antibodies as indicated. (**b**) Expression analysis of GATOR1 genes. HEK293 cells, transiently transfected with scramble shRNA or shRNAs targeting *DEPDC5*, *NPRL2* and *NPRL3* genes for 48 h prior to RNA isolation. Gene expression was normalized relative to the housekeeping gene, *GAPDH*. *APRT* (housekeeping gene) was used as an additional control. The dashed line indicates relative gene expression in pcDNA3 transfected cells. Bar diagrams represent SE of n = 3 independent experiments. ***p<0.001. Statistical differences between groups were determined using ANOVA with Tukey's post-hoc test. (**c**) The histogram shows the cell size distribution of HEK cells transfected with shRNAs targeting *DEPDC5*, *NPRL2* and *NPRL3* genes for 48 h. (**d**) HEK293 cells, transiently transfected with scramble (Scr*RNA*) or shRNAs targeting *DEPDC5*, *NPRL2* and *NPRL3* genes for 48 h, were amino acid starved for 4 h prior to immunoblotting for the indicated proteins.

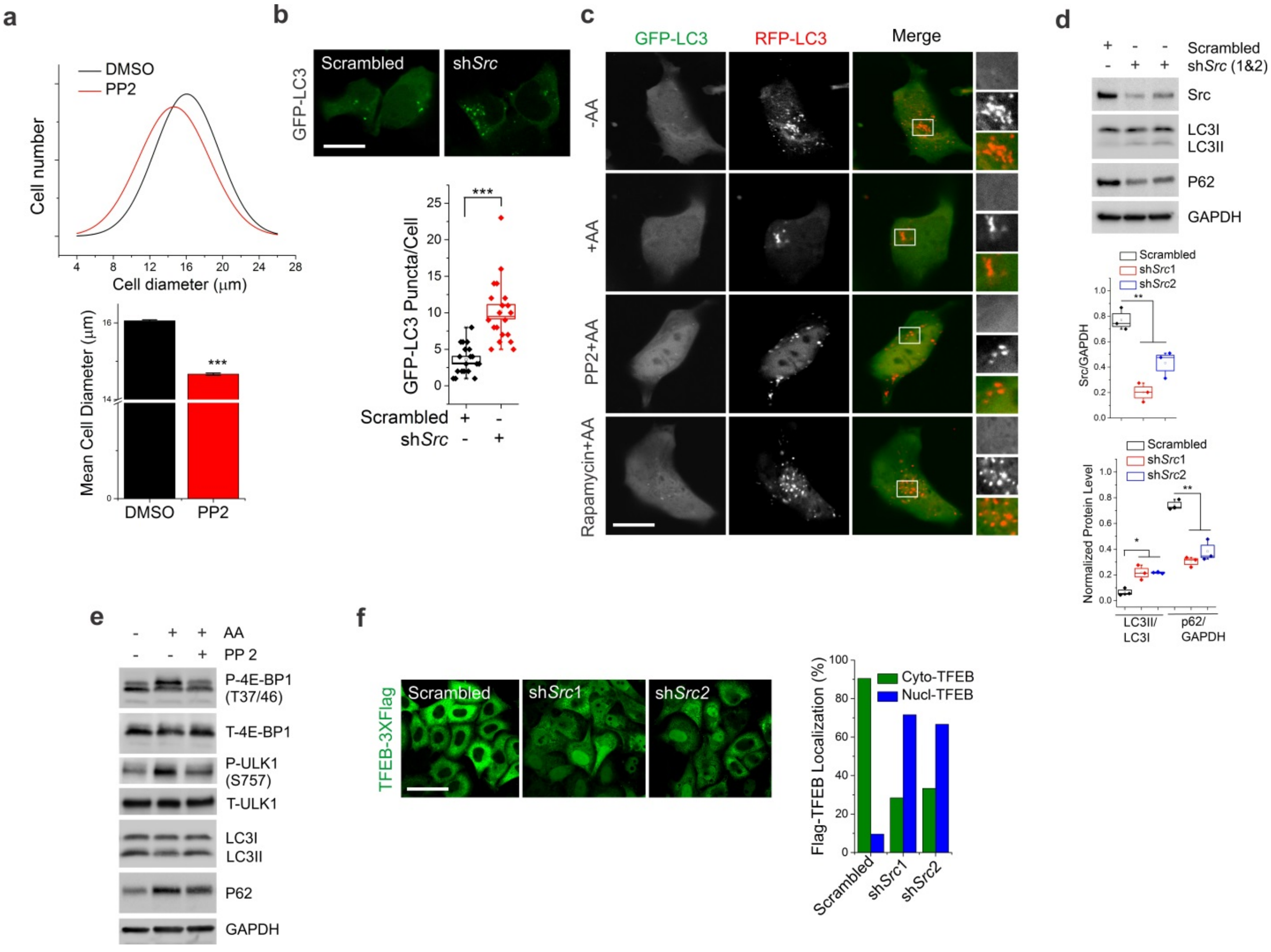

Supplementary Figure 5 (**a**) Histogram showing the cell size distribution of HEK cells treated with DMSO or PP2 (5 µM for 24 h). The bar diagram represents the mean diameter of at least 1x10^4 cells ± SEM. ***p<1x10^{-6}. (**b**) SH-SY5Y cells were co-transfected with GFP-LC3 and scrambled shRNA or sh*Src*. Live-cell imaging was performed to monitor lipidated-LC3 puncta formation. The box plots represent quantified data of lipidated-LC3 puncta from at least 15 cells. ***p<0.001. Statistical differences between groups were determined using ANOVA with Tukey's post-hoc test. Bar indicates 40 µm. (**c**) SH-SY5Y cells, transiently transfected with GFP-RFP-LC3, starved of serum (16 hr) and amino acids (4 hr) were treated with vehicle (DMSO), PP2 (10 µM) or rapamycin (600 nM) for the last two hours of starvation and then stimulated with amino acids (30 min). Live-cell imaging was performed to monitor GFP-RFP-fluorescence. Representative cells are shown where insets show selected fields that were magnified by a factor of four and their overlays. Bar indicates 40 µm. (**d**) SH-SY5Y cells transiently transfected with scrambled or sh*Src* prior to immunoblotting for the indicated proteins. The box plots represent SE of n = 3 independent experiments. *p<0.05 and **p<0.01. Statistical differences between groups were determined using ANOVA with Tukey's post-hoc test. (**e**) HEK cells starved of serum (16 hr) and amino acids (4 hr) were treated with vehicle (DMSO) or PP2 (10 µM) for the last two hours of starvation and then stimulated with amino acids (30 min). Immunoblot analyses were used to measure the levels of the indicated proteins and phosphorylation states. (**f**) HeLa cells, stably transfected with Flag-TFEB, were transiently transfected with scrambled or sh*Src*. Cells were immunostained with Flag (green) for the analyses of cytosolic and nuclear localization of TFEB. Bar indicates 80 µm. GAPDH was used as a loading control in all immunoblot assays.

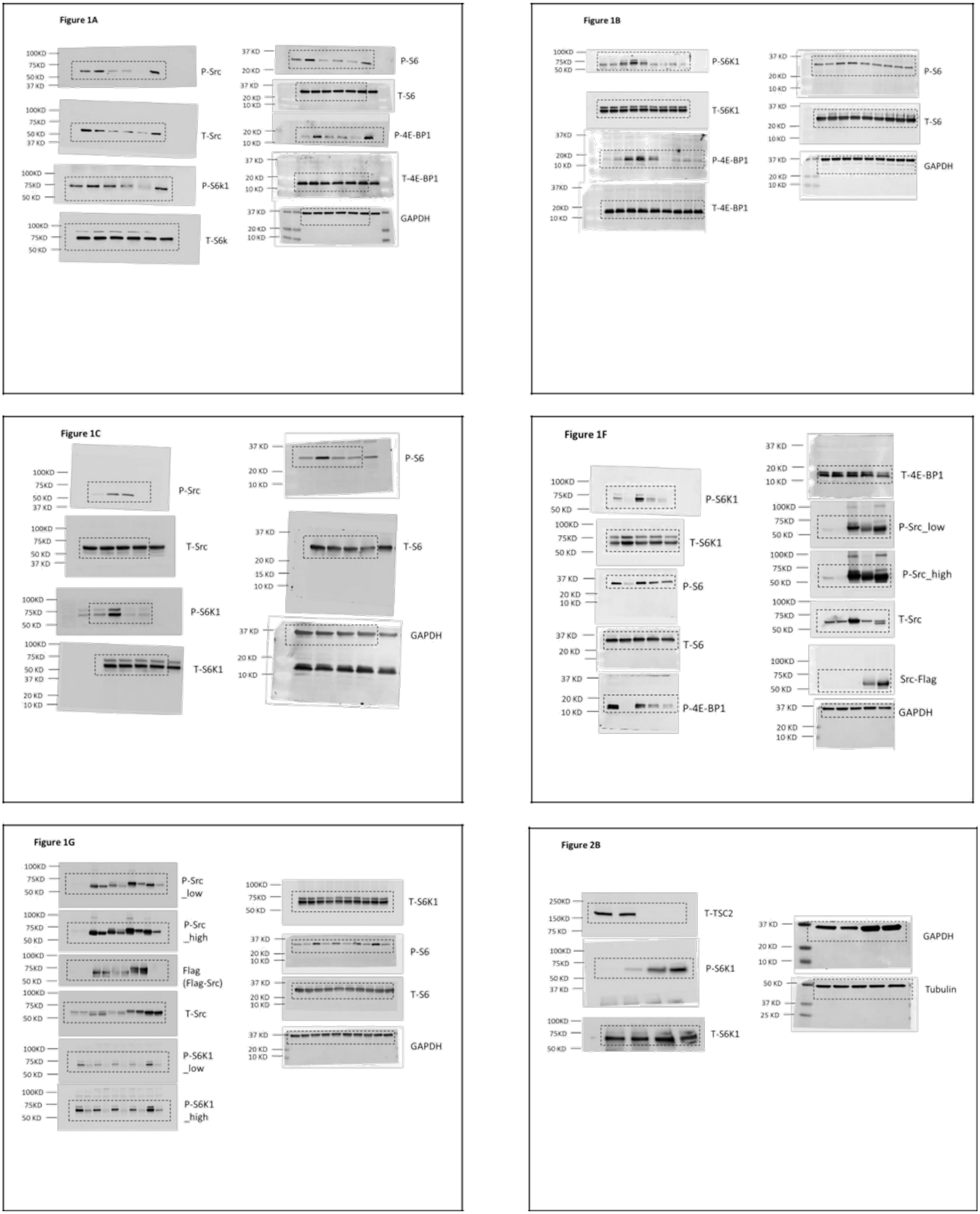

Supplementary Fig. 6. Uncropped gel images.

Figure 2C

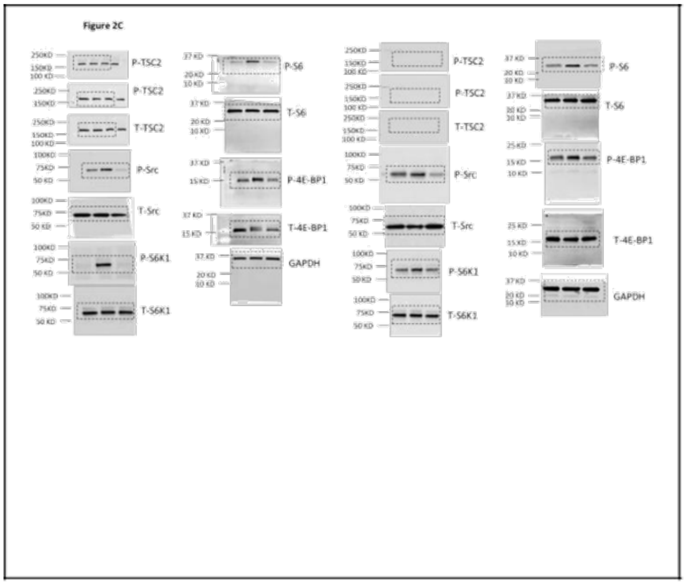

Figure 2D

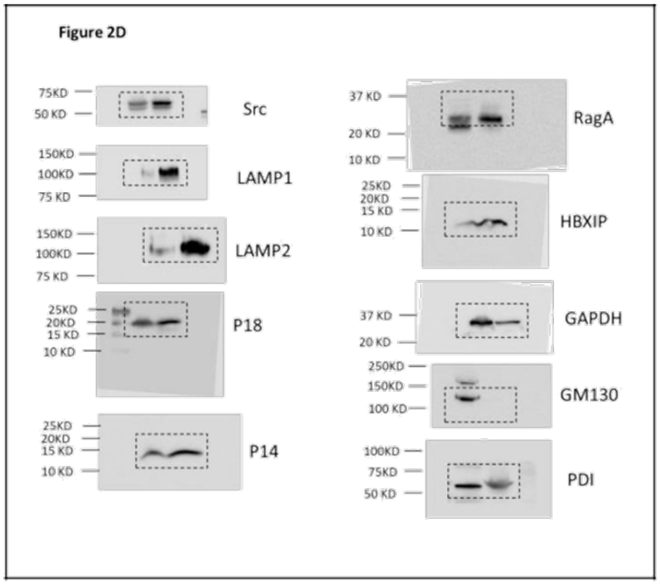

Figure 2G

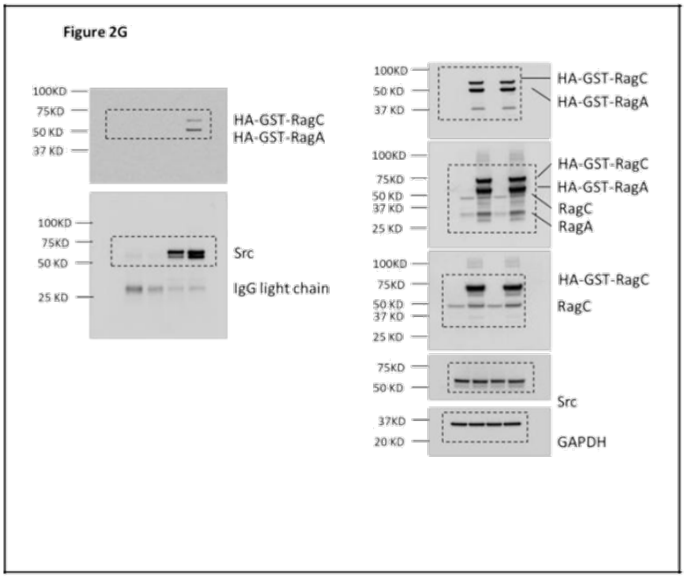

Figure 2H

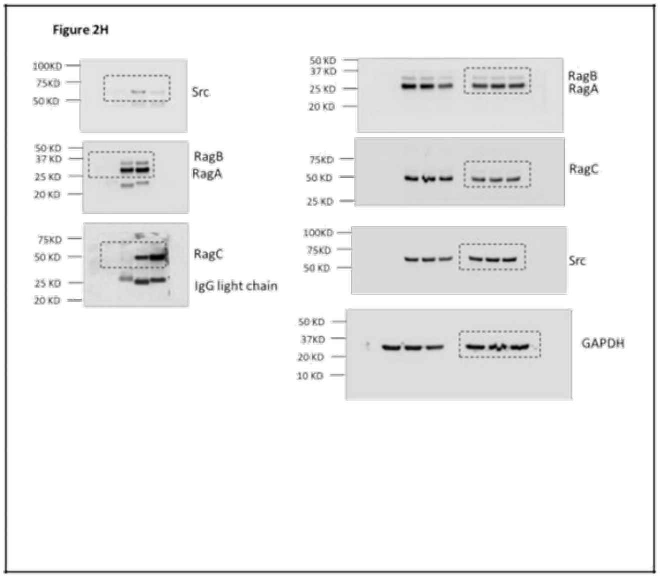

Figure 2I

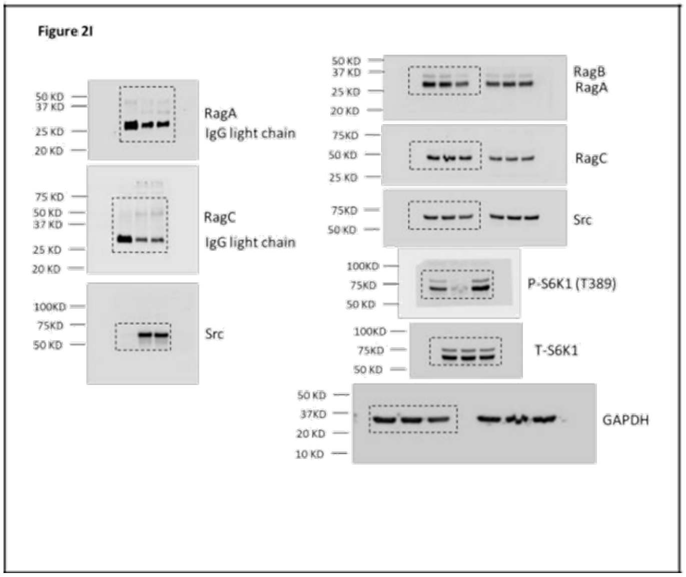

Figure 3A

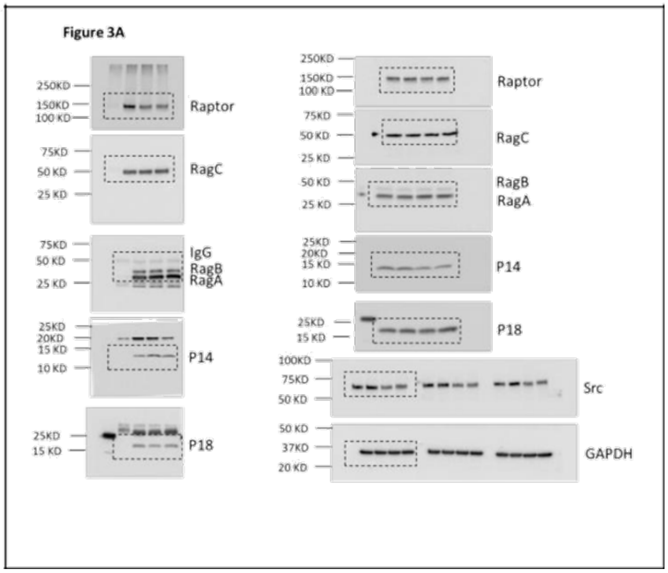

Supplementary Fig. 6--continued. Uncropped gel images.

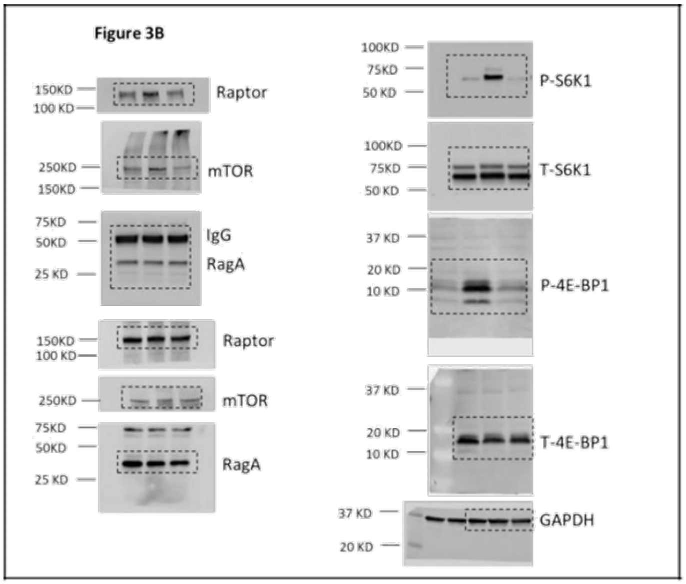

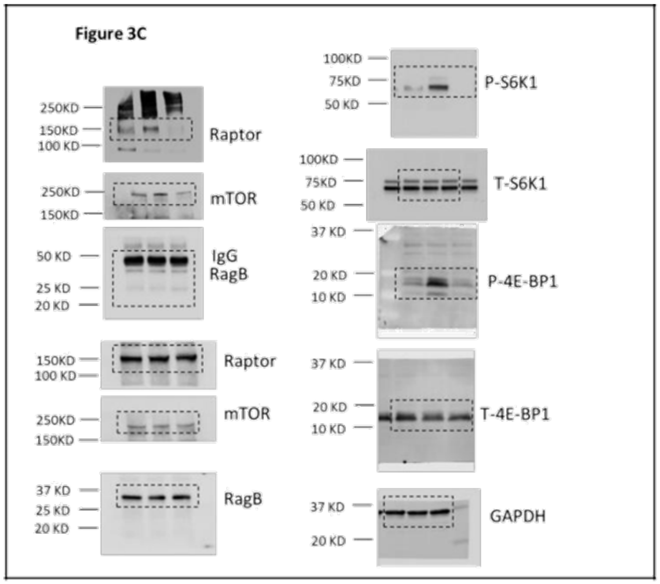

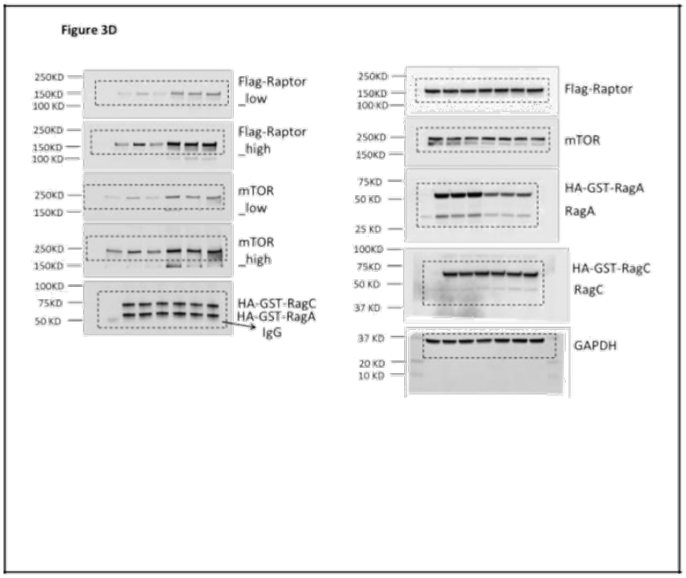

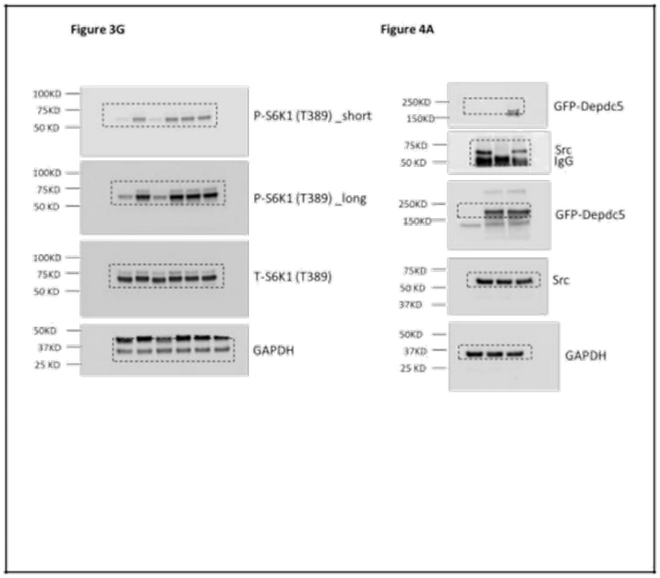

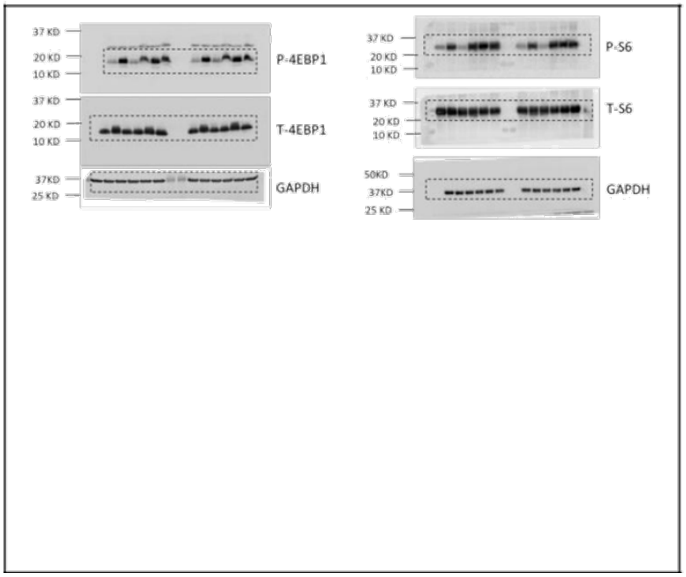

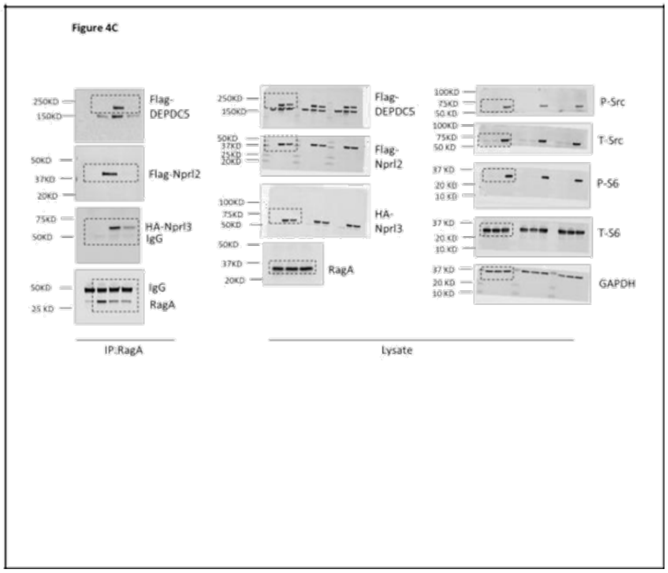

Supplementary Fig. 6--continued. Uncropped gel images.

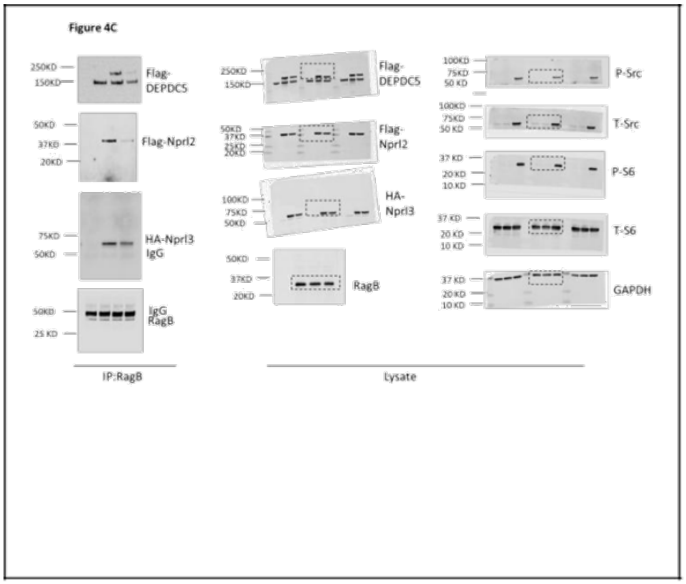

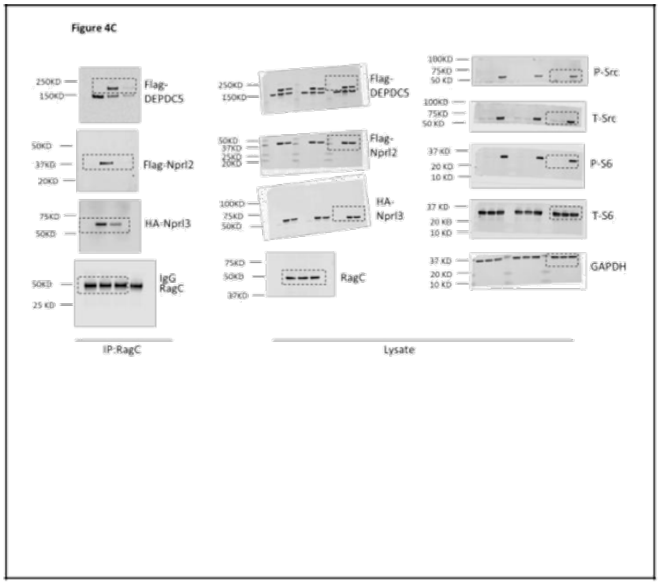

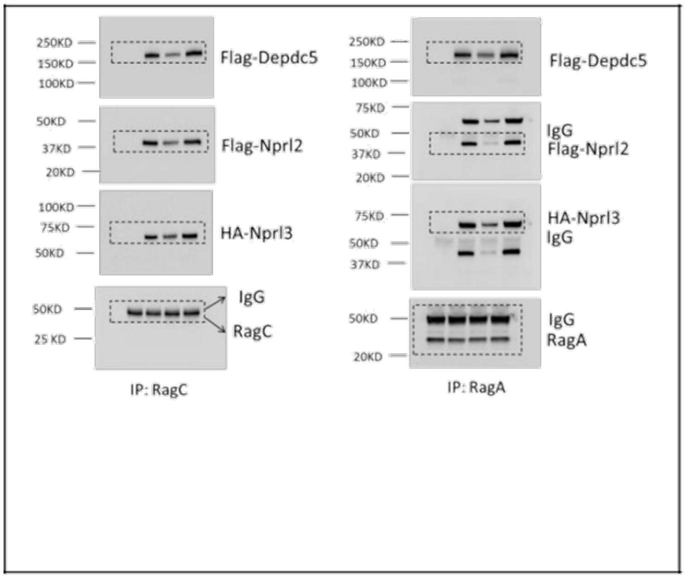

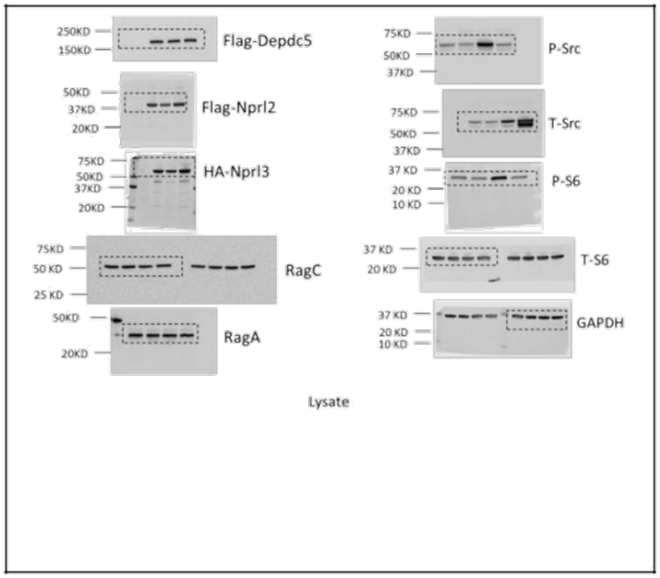

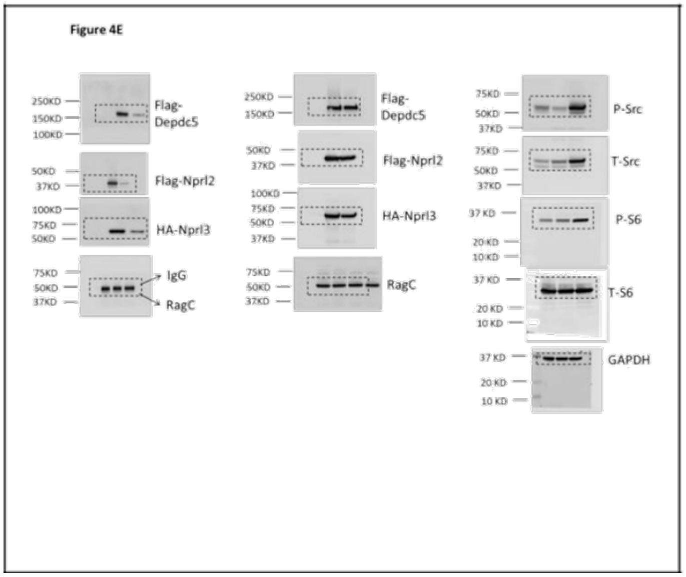

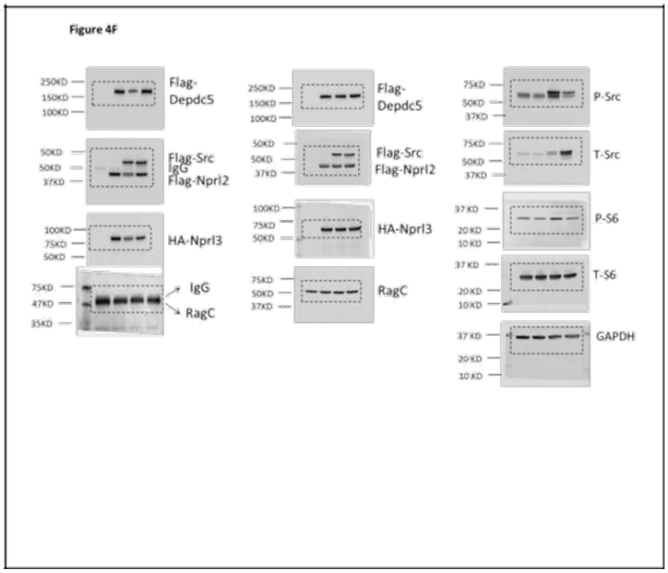

Supplementary Fig. 6--continued. Uncropped gel images.

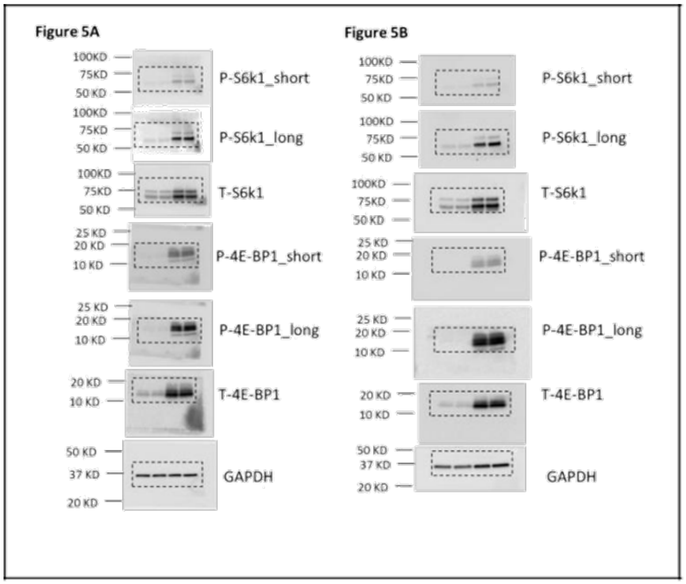

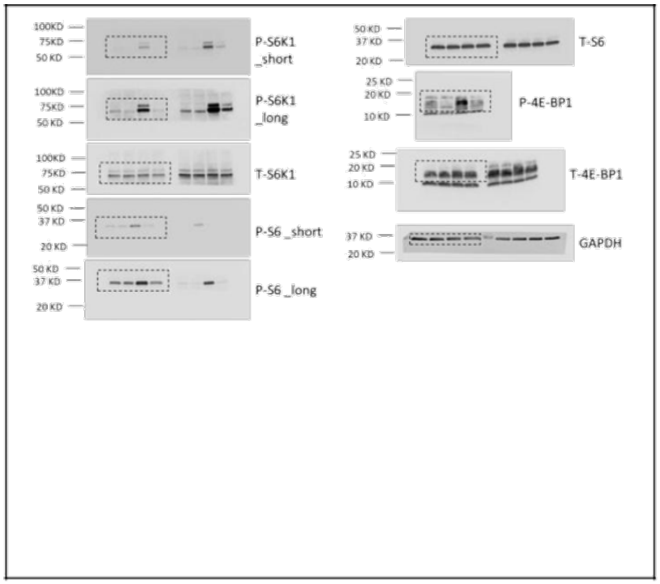

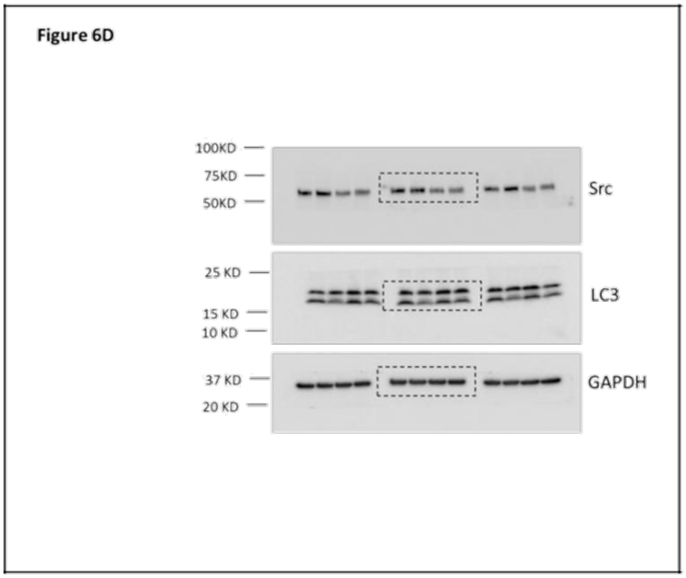

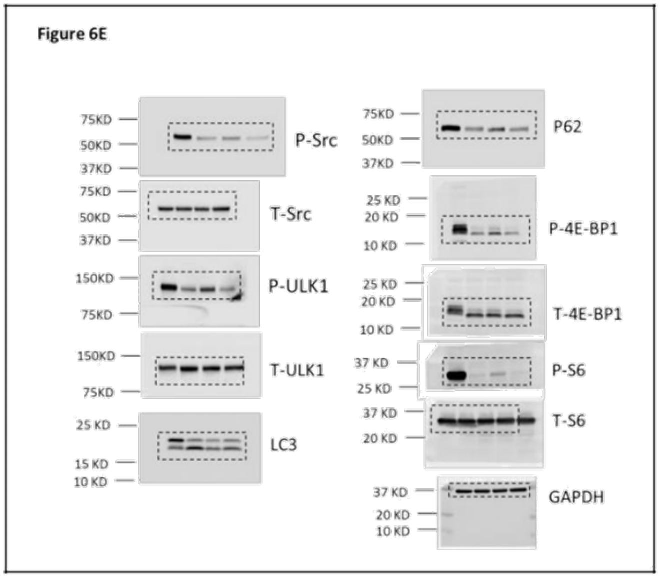

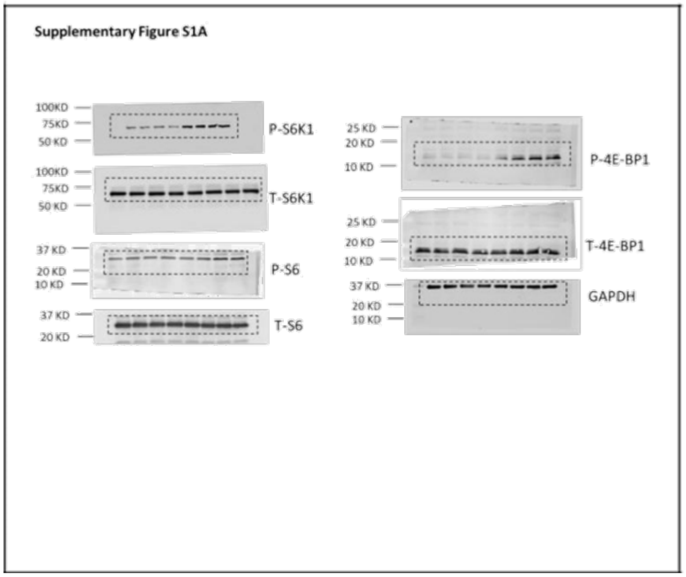

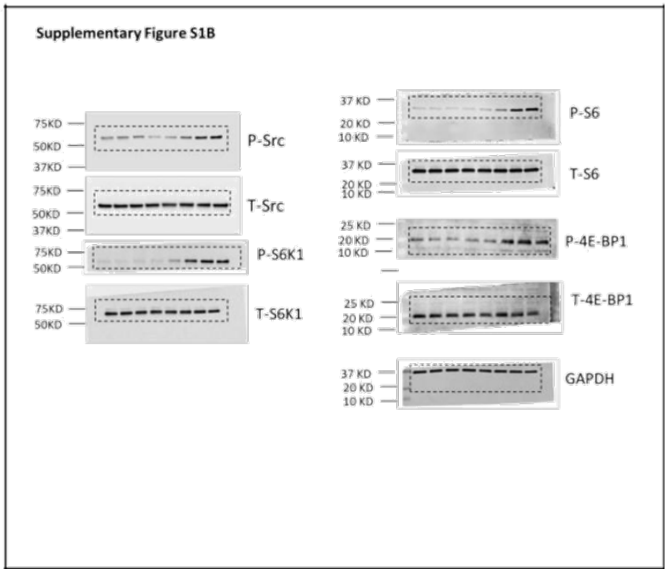

Supplementary Fig. 6--continued. Uncropped gel images.

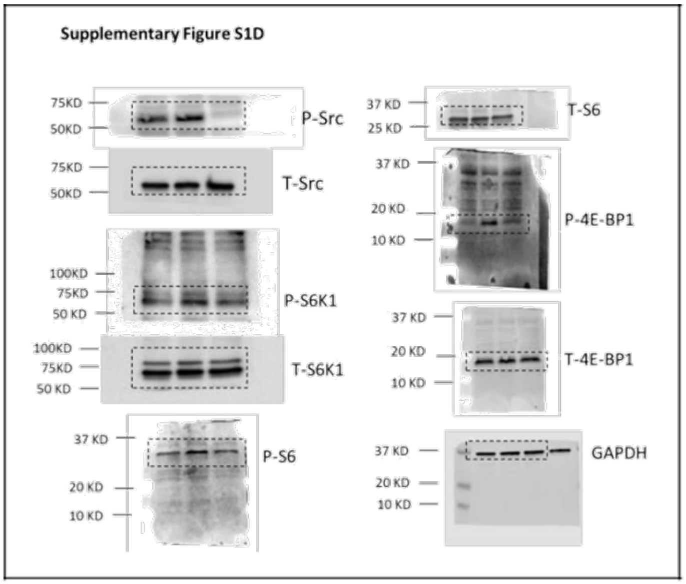

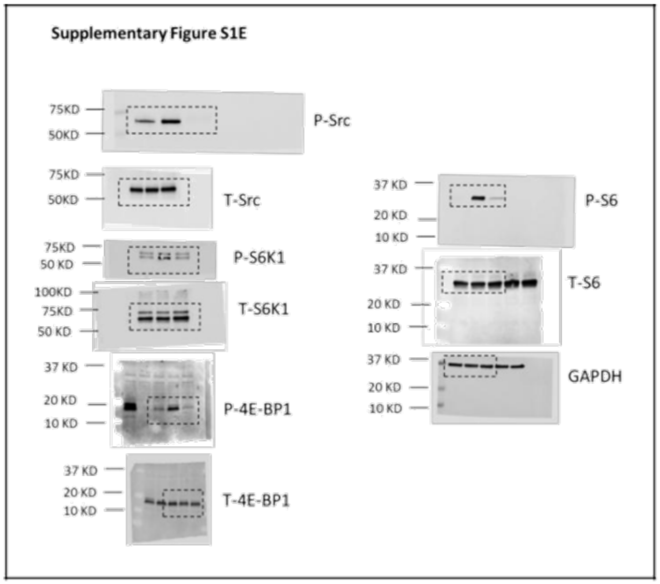

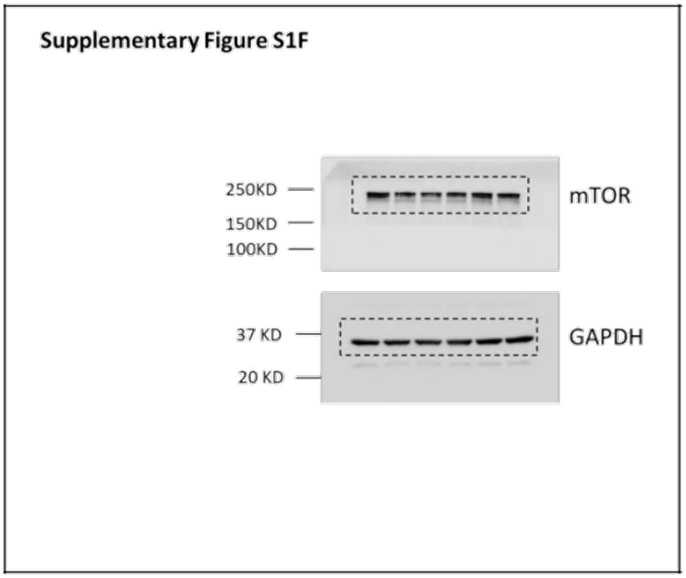

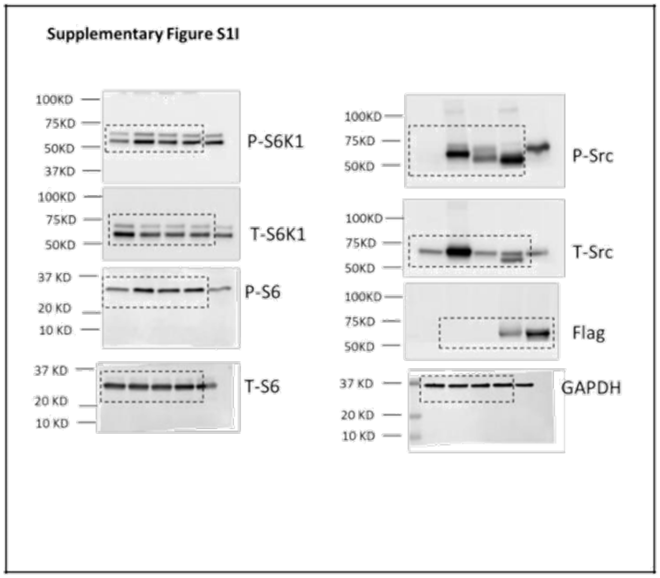

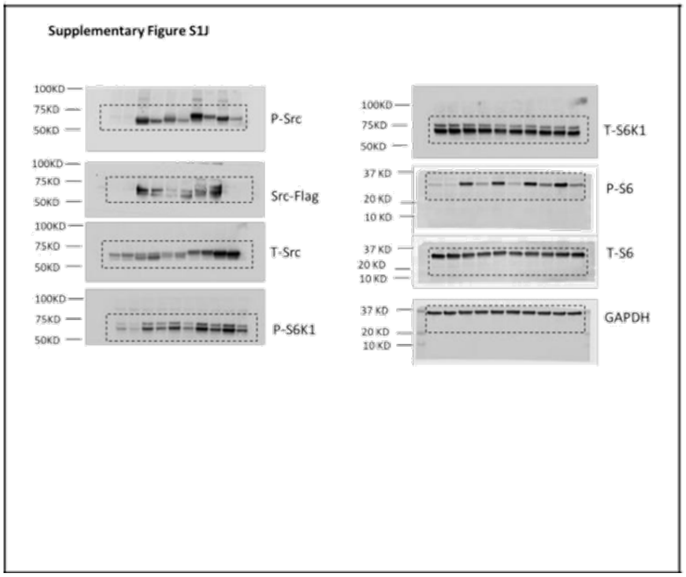

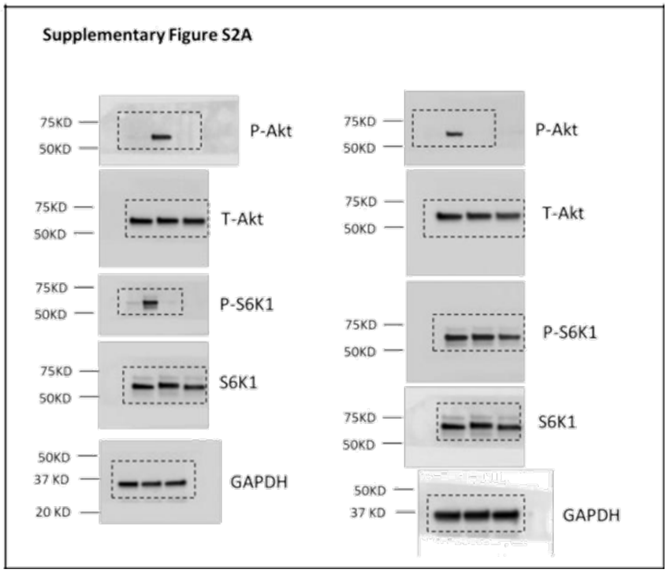

Supplementary Fig. 6--continued. Uncropped gel images.

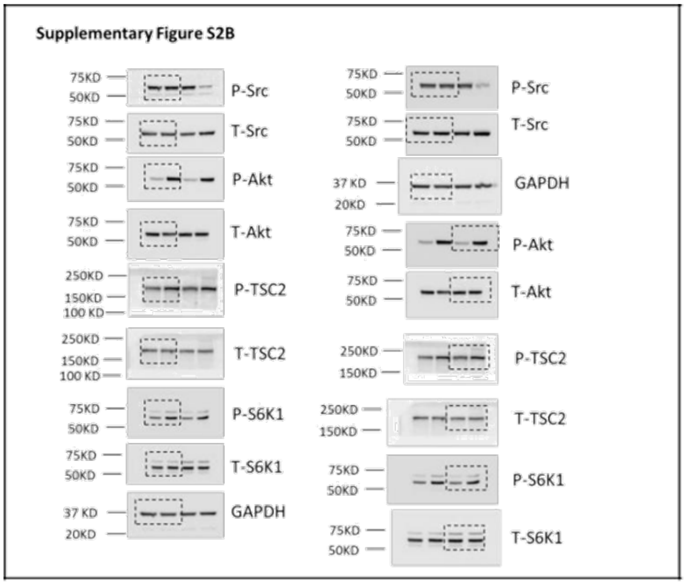

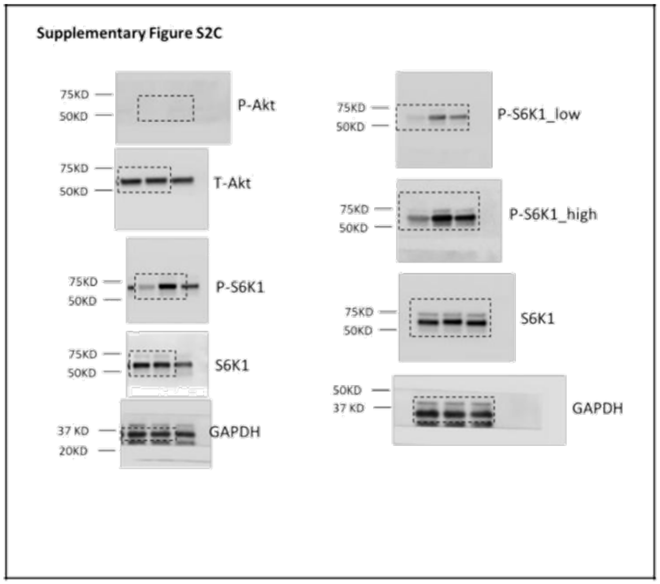

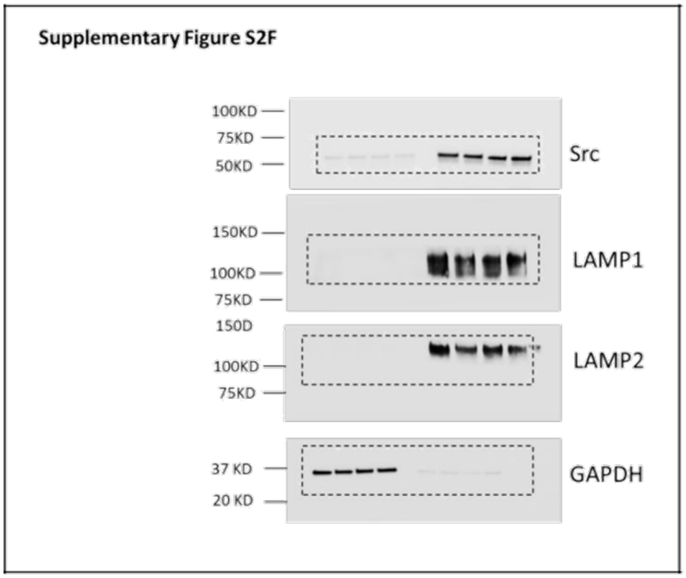

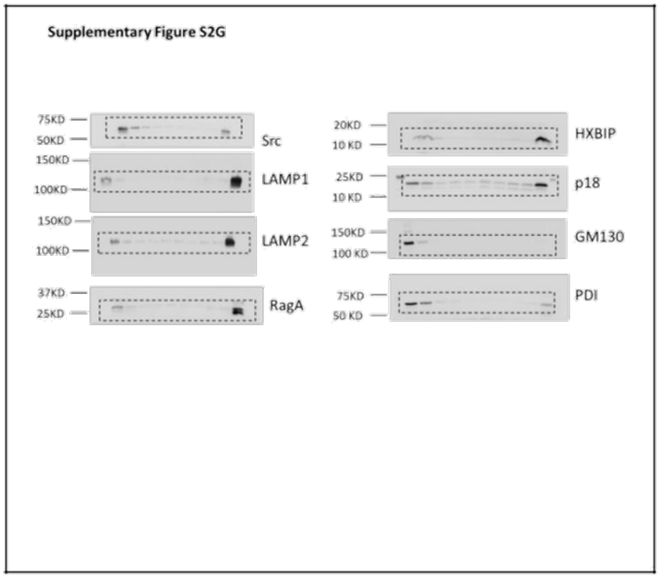

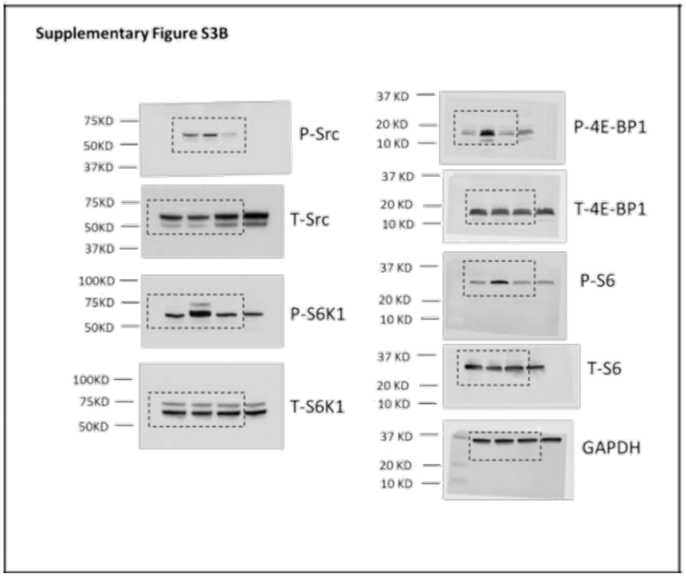

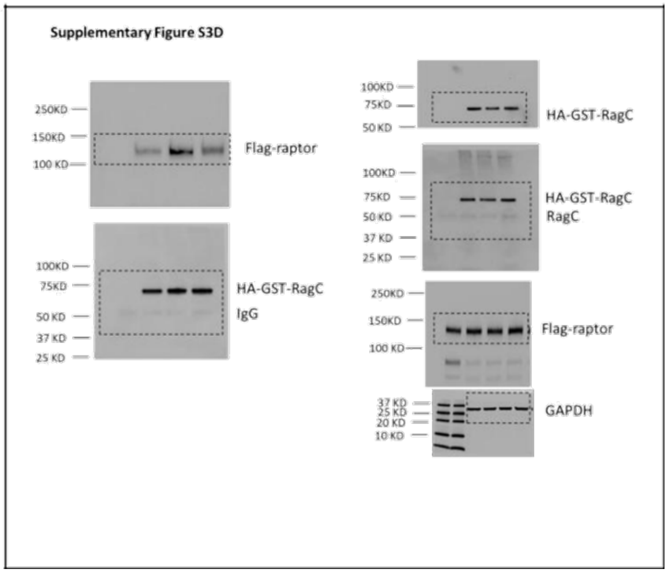

Supplementary Fig. 6--continued. Uncropped gel images.

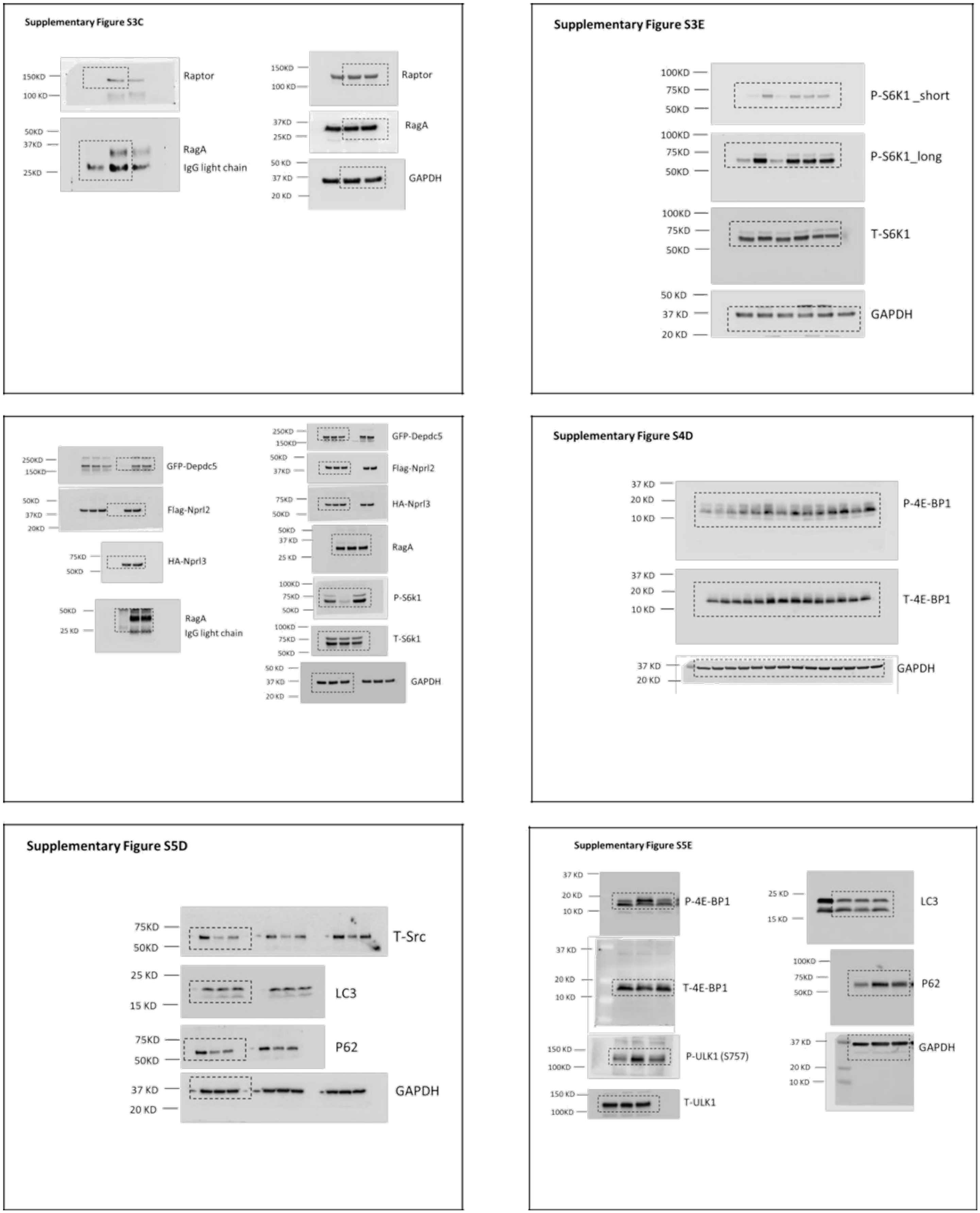

Supplementary Fig. 6--continued. Uncropped gel images.

Supplementary Table 1. Recipe of 10X amino acids mix.

Amino acids	Amount (gm)
Adenine Hemisulfate	0.2
Uracil	0.2
L-Tyrosine	0.2
L-Tryptophan	1
L-Histidine-HCL	1
L-Arginine-HCL	1
L-Leucine	1
L-Isoleucine	1
L-Phenylalanine	1
L-Glutamic Acid	1
L-Aspartic Acid	1
L-Valine	3
L-Threonine	4
L-Serine	8

Supplementary Table 2. Antibody Information.

Name of the antibody	Company	Catalog number	Dilution
P-S6K1 (T389)	Cell signaling	9234S	1:500
T-S6K1	Cell signaling	9202S	1:1000
P-S6 (S235/236)	Cell signaling	2211S	1:1000
P-S6 (S240/244)	Cell signaling	2215S	1:1000
T-S6	Cell signaling	2217S	1:1000
P-4E-BP1 (T37/46)	Cell signaling	9459S	1:1000
T-4E-BP1	Cell signaling	9452S	1:1000
GAPDH	Millipore	MAB374	1:1000
p-mTOR (S2448)	Cell signaling	5536S	1:300
mTOR (WB)	Cell signaling	2972S	1:500
mTOR (IF)	Cell signaling	2983P	1:100
LAMP2	Santa cruz	SC-18822	1:1000
Flag (mouse)	SIGMA	F3165	1:1000
Flag (rabbit)	SIGMA	F7425	1:1000
P-Src (Y416 for mouse and Y419 for human)	Cell signaling	2101S	1:500
T-Src	Cell signaling	2108S	1:1000
P-TSC2 (S939)	Cell signaling	3615S	1:500
P-TSC2(T1462)	Abcam	Ab109403	1:500
T-TSC2 (WB)	Abcam	Ab32554	1:1000
T-TSC2 (IF)	Cell signaling	4308	1:100
Tubulin	Santa cruz biotechnology	SC-23948	1:500
LAMP1 (IF)	Santa cruz biotechnology	SC-19992	1:200
P18	Cell signaling	8975S	1:500
P14	Cell signaling	8145S	1:500
HBXIP	Cell signaling	14633S	1:500
RagA	Cell signaling	4357S	1:500
RagB	Cell signaling	8150S	1:500
RagC	Cell signaling	9480S	1:1000
GM130	Abcam	Ab52649	1:500
PDI	Santa cruz biotechnology	SC-20132	1:300
Raptor	Cell signaling	2280	1:500
HA (IP) (rabbit)	Bethyl Laboratories	A190-108A	1:50
HA(WB&IF) (mouse)	Abcam	Ab130275	1:1000(WB), 1:200(IF)
P-ULK1 (S757)	Cell signaling	6888S	1:500
T-ULK1	Cell signaling	8054S	1:1000
LAMP1 (WB)	Cell signaling	3243S	1:1000
P62	BD transduction Laboratories	610833	1:500
LC3	Cell signaling	2775S	1:500
GFP	Cell signaling	2965S	1:1000
Tom20	Santa cruz biotechnology	Sc-11415	1:500 (WB)<br>1:100 (IF)
PMP70	SIGMA	SAB4200181	1:1000 (WB)<br>1:100 (IF)
KDEL	Abcam	Ab12223	1:100 (IF)
IgG	Santa cruz biotechnology	Sc-2027	Concentration equal to the IP antibody
